# A molecular mechanism for the enzymatic methylation of nitrogen atoms within peptide bonds

**DOI:** 10.1126/sciadv.aat2720

**Published:** 2018-08-24

**Authors:** Haigang Song, Niels S. van der Velden, Sally L. Shiran, Patrick Bleiziffer, Christina Zach, Ramon Sieber, Aman S. Imani, Florian Krausbeck, Markus Aebi, Michael F. Freeman, Sereina Riniker, Markus Künzler, James H. Naismith

**Affiliations:** 1 Biomedical Sciences Research Complex, North Haugh, University of St. Andrews, Fife KY16 9ST, UK.; 2 Division of Structural Biology, Wellcome Trust Centre of Human Genomics, Roosevelt Drive, Oxford OX3 7BN, UK.; 3 Institute of Microbiology, Department of Biology, Eidgenössische Technische Hochschule (ETH) Zürich, Zürich, Switzerland.; 4 Laboratory of Physical Chemistry, Department of Chemistry and Applied Biosciences, Eidgenössische Technische Hochschule (ETH) Zürich, Zürich, Switzerland.; 5 Department of Biochemistry, Molecular Biology, and Biophysics, and BioTechnology Institute, University of Minnesota–Twin Cities, St. Paul, MN 55108, USA.; 6 State Key Laboratory of Biotherapy, Sichuan University, Chengdu, China.; 7 Research Complex at Harwell, Rutherford Laboratory, Didcot, Oxfordshire OX11 0FA, UK.

## Abstract

The peptide bond, the defining feature of proteins, governs peptide chemistry by abolishing nucleophilicity of the nitrogen. This and the planarity of the peptide bond arise from the delocalization of the lone pair of electrons on the nitrogen atom into the adjacent carbonyl. While chemical methylation of an amide bond uses a strong base to generate the imidate, OphA, the precursor protein of the fungal peptide macrocycle omphalotin A, self-hypermethylates amides at pH 7 using *S*-adenosyl methionine (SAM) as cofactor. The structure of OphA reveals a complex catenane-like arrangement in which the peptide substrate is clamped with its amide nitrogen aligned for nucleophilic attack on the methyl group of SAM. Biochemical data and computational modeling suggest a base-catalyzed reaction with the protein stabilizing the reaction intermediate. Backbone N-methylation of peptides enhances their protease resistance and membrane permeability, a property that holds promise for applications to medicinal chemistry.

## Teaser

We report the structure of a fungal enzyme that activates the amide bond, allowing nucleophilic chemistry.

## INTRODUCTION

The defining feature of the peptide bond is the delocalization of the lone pair of electrons on the nitrogen atom. As a consequence, the N–C bond has partial double bond character, making it shorter, stronger, and rotationally locked—factors that shape protein structure—when compared to a normal single bond. While the lone pair in sp^3^-hybridized nitrogen is highly nucleophilic, the delocalized lone pair of the sp^2^-hybridized amide nitrogen has no nucleophilic character. Breaking conjugation to make the nitrogen nucleophilic requires overcoming the barrier to rotation around the C–N bond estimated at 77 kJ mol^−1^ (*1*), and that, alongside the p*K*_a_ (where *K*_a_ is the acid dissociation constant) [estimated in dimethyl sulfoxide (DMSO) > 20 (*2*)], presents a formidable barrier to reaction. Consequently, the chemical methylation of an amide bond typically deprotonates the nitrogen using strong bases, but this can epimerize amino acids, remove protecting groups, or lead to other unwanted side reactions (*1*). Many natural products, including those from nonribosomal peptide synthetase (NRPS) pathways and ribosomally synthesized and posttranslationally modified peptide (RiPP) pathways, contain N-methylated amides. However, it is the free amino terminus of peptides or amino acid building blocks that is methylated before peptide bond formation (*3*–*6*). Synthetic approaches commonly use methylated building blocks mirroring biology’s strategy seen in NRPSs; however, in solid-phase peptide synthesis, methylated amino acids pose significant technical challenges (*7*). The drive for methylated peptides comes from the observation that amide methylation results in a pharmaceutically useful increase in cell membrane permeability and oral availability (*8*, *9*), illustrated by cyclosporine A. The ability to easily methylate complex peptides could transform the field of peptide therapeutics by allowing access to beyond the “rule of five” compounds (*10*) that are capable of disrupting protein-protein interactions.

**Fig. 1:**
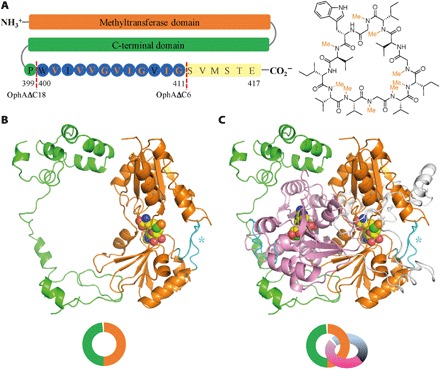
Omphalotin A and OphA. (**A**) The primary structure of OphA protein consists of a methyltransferase domain (orange), the clasp domain (green), and the C-terminal substrate peptide (blue). The dashed red line indicates the C termini of the OphAΔC18 and ΔC6 constructs. Omphalotin A derives from N to C self-methylation of the 9 of 12 possible amides within this sequence. (**B**) OphAΔC18 monomer shown as a cartoon with SAM cofactor shown as space fill (oxygen, red; nitrogen, blue; carbon, yellow; and sulfur, green). The N-terminal methyltransferase (orange) is connected to a C-terminal domain (green) forming a ring. An extended loop (in cyan and denoted by an asterisk) sits above the active site. (**C**) The dimer has a pseudo interlocking ring (catenane) arrangement, the other monomer is colored pink (N-terminal methyltransferase domain) and gray (C-terminal clasp domain). The clasp terminal domain wraps around the loops that cover the active site of the other monomer.

Omphalotin A is a fungal peptide macrocycle comprising of 12 residues of which 9 are methylated at the backbone amide (Fig. 1A) (*11*), and it is toxic to the plant parasitic nematode *Meloidogyne incognita*. Amide methylation was assumed to arise from an NRPS pathway (*12*). The discovery that omphalotins are ribosomally synthesized (*12*) means that nature has evolved a catalytic strategy for amide methylation of already formed peptide bonds. Omphalotin A derives from the C terminus of the OphA protein, and it is the OphA protein that methylates its own C terminus using *S*-adenosyl methionine (SAM) (*12*). The C terminus is subsequently cleaved off and macrocyclized to give the final product; 1 mol of enzyme gives 1 mol of product (*12*, *13*). The biological chemistry of amides is limited, and the most well-known example is the N-glycosylation of proteins in which an oligosaccharide is transferred to the side-chain amide nitrogen of the Asn in the Asn-X-Ser/Thr consensus sequence by the enzyme oligosaccharyltransferase (*14*). The current model for the reaction mechanism, based on structural and biochemical data of the bacterial oligosaccharyltransferase PglB (*15*, *16*), proposes that two metal-bridged aspartic acids form strong hydrogen bonds to the primary amide protons, resulting in the nitrogen twisting out of conjugation and thus reverting to sp^3^ hybridization and regaining its nucleophilicity. Direct methylation of an amide occurs in the ansamitocin pathway (non-RiPPs) by an unknown mechanism (*17*).

Here, we report the structure of the peptide amide methyltransferase OphA including complexes with cofactor [SAM and *S*-adenosyl homocysteine (SAH)], cofactor analog, substrate, and product. Site-directed mutagenesis and structural biology have identified residues essential in anchoring the substrate amide adjacent to SAM. Biochemical analysis supports a base-catalyzed mechanism. With these data and guided by quantum mechanical (QM) calculations, we propose that OphA uses a mechanism in which the enzyme facilitates the removal of the amide proton and stabilizes the resulting negative charge, thereby activating the amide bonds for methylation.

### Structure of OphA

**Fig. 2:**
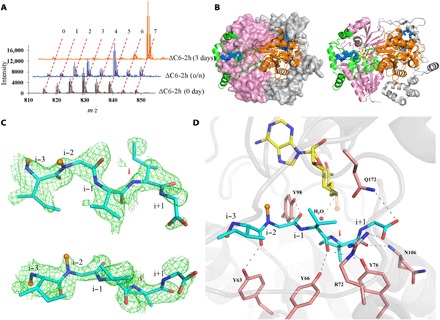
Co-complexes of OphAΔC6. (**A**) Mass spectrometry (MS) analysis of typically digested peptides shows that OphAΔC6 can be isolated in a partially methylated form (after a 2-hour induction in *E. coli*), which, upon incubation with SAM in vitro [overnight (o/n) and after 3 days], becomes increasingly methylated. (**B**) The C-terminal substrate peptide (colored dark blue) in monomer A inserts into the active-site monomer B (and vice versa; the substrate peptide of monomer B is cyan). The dimer structure is otherwise colored as in Fig. 1C. The substrate peptide binding site forms a tunnel. The orientation is rotated 180° from Fig. 1C. (**C**) A zoomed-in view (i−3 to i+1 range) of simulated-annealing Fo-Fc omit electron density contoured at 2σ for the OphAΔC6-SAH/M structure. The electron density shows methylated amides. Full electron density for all structures is shown in fig. S3. Carbons are shown in cyan, oxygen in red, nitrogen in blue, and methylation sites in orange. (**D**) The OphAΔC6-SAH/M complex shows Ile^410^, which is not methylated, opposite SAH/M (denoted position i) and so represents a product complex. Residues that have already been methylated are N-terminal to position i and shown with an orange ball and stick. The substrate peptide (shown as sticks) makes a number of hydrogen bonds with conserved residues.

Automethylation of OphA (Fig. 2A) occurs during heterologous overexpression in *Escherichia coli*, and the extent of methylation correlates with the time and temperature of induction but is a slow reaction (*12*, *13*). The originating fungus is long-lived (*11*), permitting the possibility of a slow native catalysis. The analysis of partially methylated OphA protein–established methylation is directional (N to C) (*12*). The full-length native protein was refractory to crystallization, but an 18-residue C-terminally truncated variant (OphAΔC18), which lacks the sequence that is methylated, was first purified, crystallized, and phased using a selenomethionine variant (Fig. 1B and table S1). Structures with SAM and SAH bound were determined by preincubating the protein with the cofactor prior to crystallization; high-performance liquid chromatography (HPLC) was used to confirm bound cofactor identity. Apart from the addition of the methyl group, the structures of the two complexes are identical [root mean square deviation (rmsd) of 0.6 Å over 684 Cα atoms]. The monomer has a compact N-terminal domain (Thr^7^ to Lys^251^), which binds SAM/SAH and is most closely related in structure to the uroporphyrinogen-III C-methyltransferase (UIIIMT) (rmsd of 2.4 Å over 197 Cα atoms; fig. S1A) (*18*), which has a similar back-to-back dimer arrangement (fig. S1B). OphA has an insertion of 14 residues (Cys^175^ to Asn^189^), which forms a loop that, along with another loop (Asp^67^ to Ser^71^), folds over the SAM-binding site and creates an extended enclosed cavity (Fig. 1B and fig. S1A). These loops partly occupy the pocket used to bind uroporphyrinogen-III in UIIIMT (*19*). SAM is bound to the protein by an extensive network of interactions (fig. S1C). Even with prolonged dialysis at 25°C in the presence of 2 M urea, we were unable to remove SAM from OphAΔC18 to generate an apoprotein (4 M urea unfolded the protein that we were unable to refold). Compared to the Pro^242^-adenosine (of SAM) interaction in UIIIMT (which binds SAM very tightly) (*19*), OphAΔC18 makes more extensive van der Waal interactions between Val^243^ and adenosine. UIIIMT operates by deprotonating the porphyrin ring with a pair of arginine residues to make a potent carbon nucleophile reaction (*19*).

The C-terminal region (Ala^252^ to Met^378^) of OphAΔC18, referred to as clasp domain hereafter, is not found in other methyltransferases and comprises extended strands and a helical bundle (Lys^322^ to Met^378^; Fig. 1B). The monomer has the appearance of a ring, with the C-terminal domain forming a thin band (Fig. 1B), which is closed by interactions between Leu^333^ and Pro^335^ from the clasp domain with Leu^27^ and Ser^31^ from the N terminus. OphA is a dimer in which the N-terminal (SAM-binding) domains are arranged facing away from the dimer interface (Fig. 1C and fig. S1B). The clasp domain of one OphAΔC18 monomer wraps around the N-terminal domain of the other monomer, giving the dimer the appearance of two interlocked rings (catenane), a structure that is rare among proteins [reviewed in (*20*)].

**Fig. 3:**
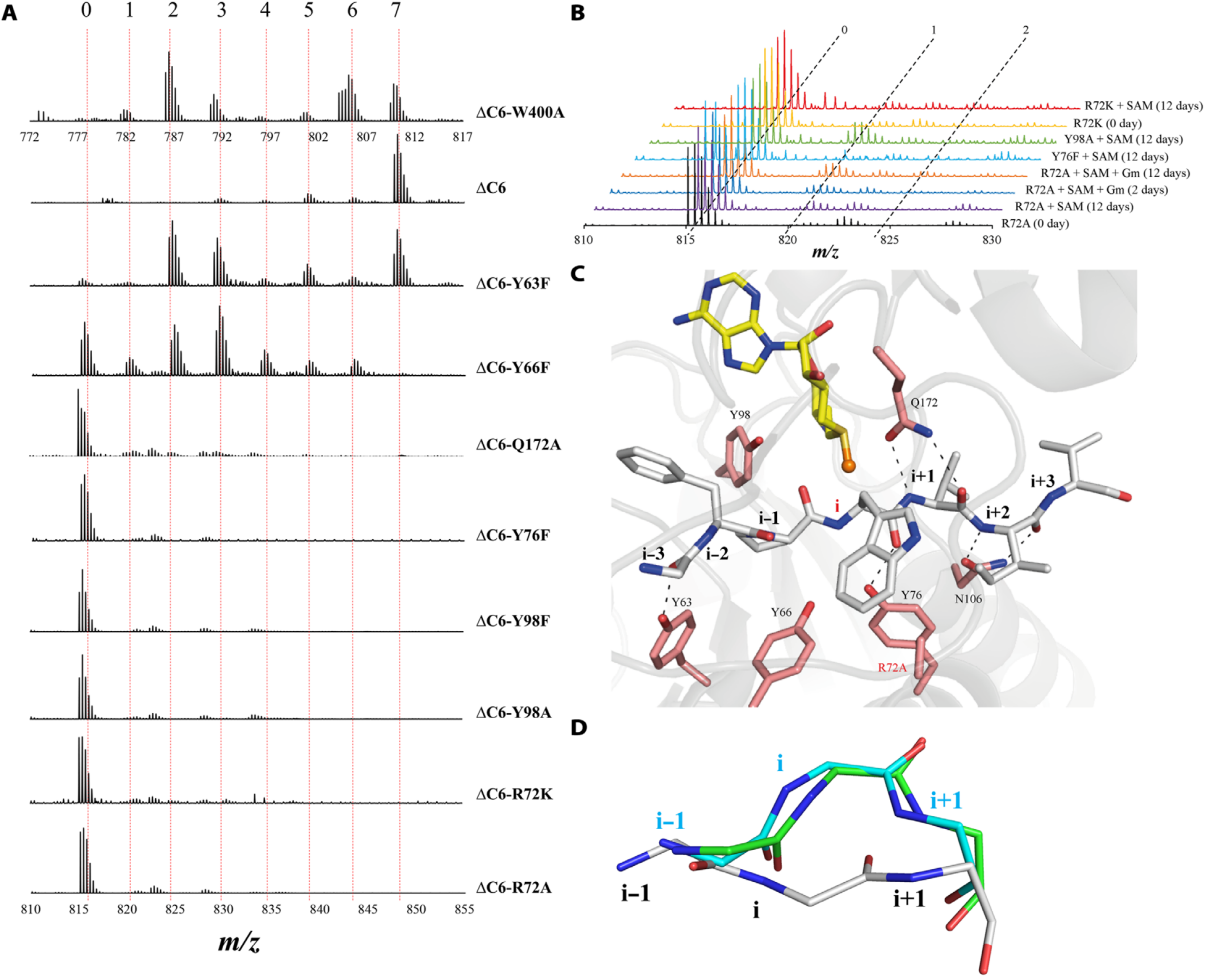
Key residues for methylation. (**A**) MS analysis of typically digested peptides of OphAΔC6 mutants after purification. Y63F, Y66F, Q172A, and W400A variants show reduced methylation, while R72A, R72K, Y76F, Y98F, and Y98A mutants show no detectable methylations at all. The W400A mutation alters the mass of the tryptic peptide and is shown with a different mass scale. (**B**) MS analysis of typically digested peptides of in vitro assay of mutants with SAM. After 12 days of incubation, Y98A shows obvious single methylation, and R72A shows methylation at the detection limit, while Y76F shows no methylation. An increase in single methylation was observed for R72A in the presence of 200 mM guanidine. (**C**) The inactive mutant R72A has the substrate peptide (i−3 to i+3 range) bound at the active site with Trp^400^ opposite SAM at position i. This substrate peptide conformation cannot undergo methylation as the amide nitrogen points away from SAM and is denoted flipped or inactive. The carbon atoms of the substrate peptide are colored gray, other atoms are colored as above. (**D**) Inactive R72A (cyan) and Y76F (green) complexes with SAH have structures in which the substrate peptide reverts to the “active” conformation. The R72A-SAM structure with the flipped/inactive conformation is shown for comparison with the same color scheme as in Fig. 3C.

A second variant of OphA, truncated at Gly^411^ (OphAΔC6; Fig. 1A), containing the core peptide, is catalytically active with methylation of up to seven residues (Fig. 3A) (*12*). Complete methylation of OphAΔC6 occurs over a similar time frame as native OphA (*12*), suggesting that this truncated version is a valid model for the full-length enzyme. Isolation and purification of OphAΔC6 after a short (2 hours) induction in *E. coli* yielded a less extensively methylated form (predominantly triply methylated; Fig. 2A). Upon further in vitro incubation with SAM, the (fully) seven-times methylated form is obtained in a time-dependent manner (Fig. 2A and fig. S2A) with an estimated *k*_cat,App_ of 0.12 hour^−1^ in bis-tris (pH 7.6) (comparable to an estimated rate in *E. coli k*_cat,App_ of 0.32 hour^−1^; fig. S2B). This similarity led us to exclude any catalytic requirement for an *E. coli* protein or metabolite. The enzyme is faster at higher pH [*k*_cat,App_ of 0.19 hour^−1^ in *N*-cyclohexyl-2-aminoethanesulfonic acid (CHES) buffer, pH 10.0]. The 1.7 Å OphAΔC6 structure determined after incubation with SAM (Fig. 2B) revealed additional density for eight residues of the core peptide (compared to OphAΔC18) bound in the active site of the other monomer (fig. S3). Electron density shows four N-methylated amides (Fig. 2C and fig. S3A). The structure is otherwise essentially identical to OphAΔC18 (rmsd of 0.6 Å over 688 Cα atoms) with no notable conformational changes of the residues that comprise the binding site. For ease of discussion, we split the protein into three sections: the N-terminal (methyltransferase) domain, the clasp domain, and the C-terminal substrate peptide (Fig. 1A). Inspection of the experimental maps reveals that while the positions of substrate backbone atoms are unambiguous, density for the side chains is suggestive of some heterogeneity in the register of the bound substrate peptide. The electron density indicates that a mixture of SAM (0.3) and SAH (0.7) is present in the crystal (fig. S3A); thus, we denote this complex OphAΔC6-SAH/M. HPLC analysis shows that the protein as isolated from *E. coli* contains almost exclusively SAH (fig. S3B) with only a small amount of SAM exchange in solution, suggesting that displacing SAH with SAM is very unfavorable when the substrate peptide is present, in line with the observed slow catalytic turnover rate in vitro. Cocrystallization with SAH does not result in any structural change, and the map now shows no density for the SAM methyl group (fig. S3A); this complex is denoted OphAΔC6-SAH. The location of the cofactor relative to the protein is identical in both the OphAΔC18 and OphAΔC6 complexes. The substrate peptide sits in the extended active-site tunnel with the amide nitrogen of Ile^410^ (we define the amide in this location as “i” hereafter) pointing toward the methyl group of SAM; the N(amide)-C(SAM)-S angle is 171°, close to the 180° required for an S_N_2 attack (Fig. 2D). The carbonyl of the residue at position i is hydrogen-bonded to the backbone amide of Asn^106^. The Ψ angle between residues i and i+1 is 0°. Consequently, the amide nitrogen atoms are arranged cis to each other, and the main chain twists 90° (Fig. 2D). The peptide carbonyl at i−3 hydrogen-bonds to the side chain of Tyr^63^, at i−2 to the side chain of Tyr^98^, and at i−1 to both Tyr^66^ and Tyr^76^. Arg^72^ hydrogen-bonds to Tyr^76^ and makes a polar contact with the carbonyl at residue i+1 (the C-terminal residue, Gly^411^). The side chain at the i−2 position sits in a well-defined pocket formed with Ile^44^, Tyr^63^, and Tyr^98^. The methyl on the amide at position i−2 makes van der Waals contacts with Val^243^, while a methyl group on the i−1 amide would contact Tyr^66^ (Fig. 2D). The side chains at i−1 and i−3 both sit in large hydrophobic pockets, while the side chains at i−4, i−5, and i−6 are exposed to solvent. In both solution and crystal, neither Ile^410^ nor Gly^411^ of OphAΔC6 is methylated. The substrate side chains do not make any specific contacts with OphA, consistent with the protein’s ability to methylate different residues (Gly, Val, Ile, Met, Thr, and Ala) (*12*, *13*).

### Identification of key residues

Sequence analysis shows that residues that bind to the substrate peptide are conserved in proteins from closely related fungi (fig. S5). Mutants Y63F, Y66F, Y76F, R72A, R72K, Y98A, Y98F, Q172A, and W400A of OphAΔC6 were expressed in *E. coli*. Y76F and Y98A led to highly aggregated protein, and only a small amount could be purified for analysis; very little folded material was obtained for Y98F, but no methylation was detected. Y63F, Y66F, Q172A, and W400A showed methylation either upon purification or after a short incubation with SAM (Fig. 3A and fig. S6) but have reduced activity. Y76F, R72A, R72K, and Y98A were unmethylated even after 3 days of overexpression in *E. coli* (Fig. 3A). During prolonged (12 days) incubation, there was some evidence for a very small amount of methylation in the Y98A mutant (>100-fold reduced in vitro activity), but not in the Y76F mutant (Fig. 3B). Careful examination of R72A supplemented with guanidine after a 12-day incubation of SAM reproducibly showed a methylated species (Fig. 3B and fig. S6). We were, however, unable to unambiguously confirm methylation of R72K at 12 days. Y63F, Y66F, Y76F, R72A, Y98A, and W400A were incubated with SAM and analyzed. HPLC analysis showed displacement of SAH by SAM in Y76F, R72A, and Y98A upon incubation (fig. S3B), whereas the other three mutants showed very little displacement by SAM. Electron density of the cofactor in the Y76F, R72A, and Y98A complexes was consistent with the HPLC analysis (fig. S3, A and B, and table S1), showing that SAM bound. In the R72A-SAM complex (rmsd of 0.7 Å over 720 Cα atoms with OphAΔC6-SAH), peptide bonds of the substrate residues i−1 and i are flipped 180° relative to the conformation observed in OphAΔC6 and Y63F-SAH. Although the side chain of residue i+1 sits in the same pocket as seen in the OphAΔC6 protein, its backbone has a different hydrogen bond arrangement (Fig. 3C). Consequently, the amide nitrogen at position i (Trp^400^) points away from SAM (Fig. 3C) and is no longer aligned for methylation. The same “flipped” arrangement of the substrate peptide is observed for SAM complexes of Y98A (Trp^400^ at position i) and Y76F (Ile^402^ at position i) (figs. S3A and S7). The flipped peptide results in Arg^72^ adopting an alternative conformation. A different conformation of Tyr^98^ is seen in inactive mutant complexes (Y76F-SAM, Y76F-SAH, and R72A-SAH). Despite the large conformational change in substrate peptide, conformations of other OphAΔC6 residues are essentially unaltered, pointing to rigidity at the active site. The electron density in the Y66F active mutant suggests that both the active and flipped substrate conformers as well as two Arg^72^ conformers are present (fig. S3A), but disorder prevented firmer interpretation. In addition, complexes R72A-SAH and Y76F-SAH show similar active conformation of the substrate peptide (Fig. 3D).

### Discounting mechanisms

Since no plausible amino acid is positioned to directly remove the proton from the amide, we considered disruption of conjugation akin to N-linked glycosyl transferase (*15*), in which two strong hydrogen bonds (thus requiring a primary amide) are made to Asn, but the OphA structure does not support this mechanism. We next considered nucleophilic addition to the carbonyl at i−1 to form a tetrahedral carbon (cf. serine protease). Among the protein side chains, TYR^66^, TYR^76^, or TYR^98^ could plausibly act as the nucleophile, (*21*). Y66F retains activity, and Tyr^76^, although within 4 Å of the carbonyl, has a Bürgi-Dunitz (44°) angle incompatible with attack, so both possibilities were discarded. Both Tyr^66^ and Tyr^76^ are, however, well positioned to stabilize the resulting tetrahedral carbon by hydrogen bonding. Tyr^98^ has plausible (but not ideal) Flippin-Lodge (−19°) and Bürgi-Dunitz (149°) angles. However, it is 5.5 Å from the carbonyl, its tetrahedral intermediate would create multiple clashes (with SAM, the side chain at position i and the main chain at position i−1), and no base is positioned to deprotonate it, leading us to exclude this mechanism. We also considered whether a water molecule could perform this attack and form a geminal diol intermediate. The reaction in the presence of H_2_^18^O shows no ^18^O incorporation in the peptide, and the structure shows no water or base appropriately positioned for this reaction, arguing against such a mechanism. Further, a geminal diol intermediate would be predicted to give rise to peptide bond cleavage at the very least as an otherwise unwanted side reaction, which was not observed. Finally, formation of a tetrahedral intermediate would seem inconsistent with the observed decreased methylation rate (kinetic isotope effect of 3) in vitro in D_2_O (fig. S9).

### Active-site geometry

**Fig. 4:**
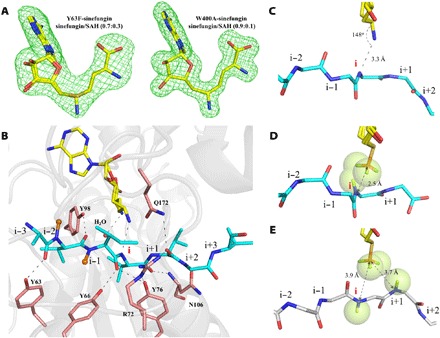
Substrate peptide and sinefungin coordination in the active site. (**A**) The simulated annealing Fo-Fc omit electron density (3σ) for sinefungin in OphAΔC6 Y63F and W400A complex. The HPLC analysis in fig. S3B shows some residual SAH remains. Carbon atoms are shown in yellow, sulfur in dark yellow, oxygen in red, and nitrogen in blue. (**B**) The OphAΔC6 W400A sinefungin structure represents a substrate complex and shows an active arrangement of the substrate peptide. The same color scheme and perspective as in Fig. 2D are used. (**C**) In both OphAΔC6 W400A (and Y63F) sinefungin complex structures, there is 3.1 to 3.3 Å hydrogen bond between the nitrogen atoms of sinefungin and the substrate amide. The arrangement of atoms is consistent with the S_N_2 attack by the amide on the cofactor. The same color scheme and perspective as in Fig. 4B are used. (**D**) Replacing SAH with SAM (in silico) shows that SAM would create van der Waal clashes with the amide; this extremely close contact is predicted to stabilize the S_N_2 transition state. (**E**) The inactive mutant R72A-SAM complex, where the anchoring interactions between substrate and enzyme are disrupted, avoids this steric clash. The enzyme does not significantly rearrange to tolerate this change in substrate conformation.

Since it was not possible to obtain a fully occupied SAM complex with any active protein, we used sinefungin (fig. S4A), an inactive SAM analog, to obtain two OphAΔC6 complexes (active mutants Y63F and W400A). HPLC analysis shows that the sinefungin/SAH ratio is 0.7:0.3 in Y63F and W400A (fig. S3B), consistent with the electron density (Fig. 4A and fig. S3A) of the cofactors, and thus, these structures are substrate complex mimics. Superposition of the Y63F sinefungin complex with OphAΔC6-SAH shows that the protein (rmsd of 0.6 Å over 363 Cα atoms), the common cofactor atoms (rmsd of 0.2 Å over 25 common atoms), and the common substrate backbone atoms (positions i−3 to i+1; rmsd of 0.7 Å over 20 common backbone atoms) are positioned near identically (fig. S4B). Superposition of both sinefungin complexes with the OphAΔC6 Y63F-SAH, OphAΔC18-SAM, and OphAΔC18-SAH complexes shows similar consistency in the protein structure, cofactor, and where present substrate positions. We conclude that the extensive hydrogen-bonding network at the active site and the catenane-like arrangement lead to a highly rigid active site. In both sinefungin complexes, the two nitrogen atoms (sinefungin and amide) approach close enough to form a hydrogen bond (distance varies between 3.1 and 3.3 Å; Fig. 4C). Using structural superposition, we noted that placing (in silico) the larger SAM molecule into the SAH complex places the methyl group approximately 2.5 Å from substrate amide nitrogen (Fig. 4D). This clash is likely the reason why we have been unable to obtain fully occupied SAM complexes with active proteins. Although close contacts have been observed between SAM and its substrate in a number of structures (*22*–*25*), OphA appears to have the shortest distance.

Mutations that delete hydrogen bonds between the substrate peptide and protein and inactivate the enzyme (R72A, Y76F, Y98F, and Y98A) have the same “inactive” or flipped conformation of the substrate peptide (fig. S7). We therefore hypothesize that, by disrupting anchoring interactions, the flipped conformation can be adopted to relieve the steric clashes between SAM and the amide that would otherwise occur (Fig. 4E). We predict that removal of the methyl group of SAM by changing to SAH would remove the “driving force” for the flipped conformation. The structures of R72A and Y76F with SAH do show the active conformation (Fig. 3D).

### Mechanism for methylation

**Fig. 5:**
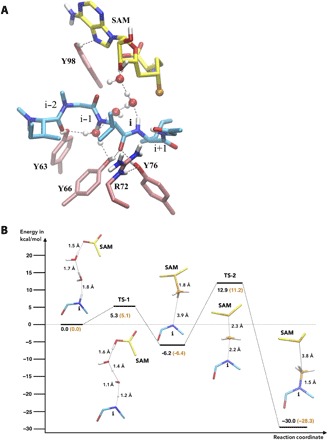
Mechanistic calculations and the role of water. (**A**) Structure of the simplified model used in the QM calculations, optimized with the B3LYP functional (*59*, *60*). Water molecules observed in the MD simulation were considered. SAM is shown in yellow, the substrate in cyan, and key residues in the binding pocket in pink. Apolar hydrogens are omitted for clarity. (**B**) Energy profile of the proposed two-step reaction obtained from the QM calculations. Results using the B3LYP functional (*59*, *60*) are shown in black, and results using the TPSSH functional are shown in orange. QM calculations require a defined location of the proton in the intermediate state for convergence. The deprotonated carboxyl group of SAM was used as the ultimate destination; however, other routes are plausible. Perfect S_N_2 alignment of the methyl group of SAM was not observed in the model system and level of theory used.

**Fig. 6:**
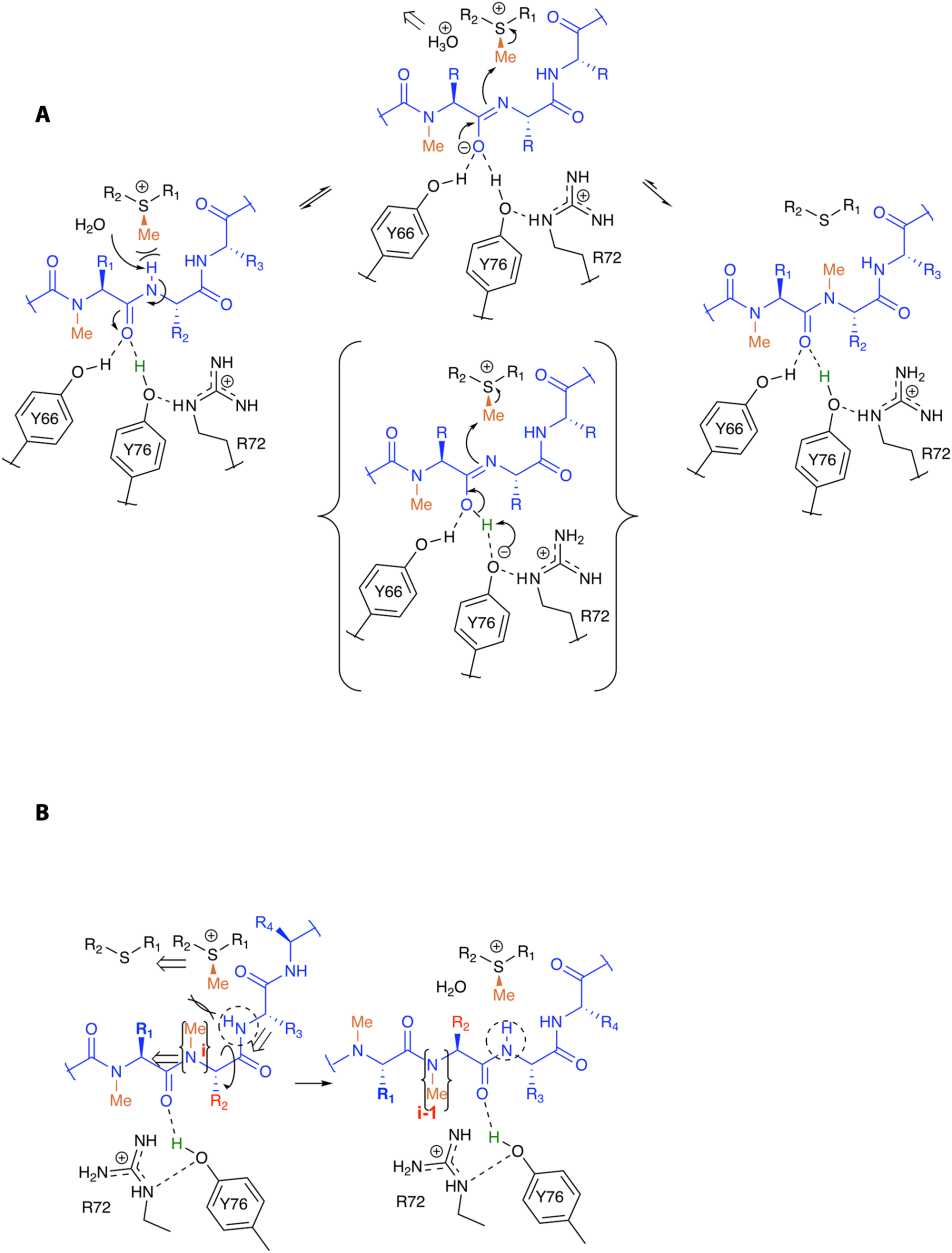
Proposed mechanism of methylation. (**A**) The conserved water molecule acts as base to remove the proton from the substrate to generate the imidate. The imidate is stabilized by hydrogen bonds to Tyr^66^ and Tyr^76^. Arg^72^ could stabilize tyrosinate and thus proton-transfer from Tyr^76^ to yield imidic acid (shown bracketed), which is essentially the formation of the imidic acid tautomer. SAM is shown in simplified form; the chemical structure is given in fig. S4A. (**B**) The displacement of SAH with SAM would create an impossible steric clash with the newly methylated amide; we propose that these clashes drive the required movement of the substrate (a 180° rotation and one residue translation). We suggest that the flipped conformation seen in Fig. 3C resembles an intermediate in the translocation process.

QM calculations show that deprotonation of the amide must occur prior to S_N_2 attack (fig. S8); notably, the educt is higher in energy than the intermediate due to the steric clash between SAM and the substrate. In all crystal structures, a water molecule, hydrogen-bonded to the amine of cofactor, is conserved 5.9 Å from the amide (Fig. 2D). Molecular dynamics (MD) and QM calculations show that this and other water molecules are mobile (Fig. 5A). Calculations show that water can function as the base (Figs. 5B and 6A) to remove the amide proton to generate the imidate. This process is similar to the base-catalyzed proton exchange of amides occurring in proteins at moderate pH (*26*). The hydrogen-bonding network of the water may enhance its basicity (Figs. 2D and 4B). The resulting negatively charged oxygen of the imidate would be stabilized by hydrogen bonds to Tyr^66^ and Tyr^76^. A barrier of 5.3 kcal/mol was calculated for formation of imidate. The observed kinetic isotope effect of around 3 and the reactions’ pH profile are consistent with such a base-catalyzed mechanism (fig. S9).

The p*K*_a_ of imidate is not known, but the p*K*_a_ of trichloro imidate has been reported as 11.2 (*27*), higher than the p*K*_a_ of 10 commonly estimated for Tyr. The Tyr^76^-Arg^72^ couple is reminiscent of the Lys-Tyr couple found in short-chain dehydrogenases/reductases (SDR) enzymes (*28*), where the positive charge stabilizes the tyrosinate anion. Proton transfer from Tyr^76^ to the oxyanion would yield the imidic acid tautomer. The imidic acid tautomer of an amide has recently been observed by neutron diffraction in an unrelated enzyme where it plays a role in catalysis (*29*).

The sp^2^ electron lone pair on the deprotonated nitrogen atom is ideally positioned for S_N_2 attack of the methyl group of SAM (Fig. 6A). Our calculations estimate a barrier of 19.1 kcal/mol for the S_N_2 attack (Fig. 5B). The heights of the barriers in the proposed mechanism are within the range typically found in enzymatic reactions (*30*). We propose that the close arrangement of reactants in OphA stabilizes the S_N_2 transition state (TS-2; Fig. 5B) (electrostatic preorganizational model) (*31*). After methylation, to reset the machinery for the next step, SAM displaces SAH. However, for SAM to fully bind, the methylated amide at position i will have to translocate to position i−1 to avoid clashing (Fig. 6B). There is an approximately 180° dihedral angle between the substrate amide nitrogen atoms at i+1 and i−1; thus, as the substrate peptide translocates through the active site, its peptide bond must also “flip.” We speculate that the flipped conformation of the substrate (seen in R72A, Y98A, and Y76F SAM complex structures) resembles an intermediate arrangement that occurs during translocation (Fig. 6B).

The amide bond is taught as the example of how resonance dominates chemical properties; the lone pair on the nitrogen atom, once conjugated, does not act as a nucleophile. It is because of this formidable kinetic barrier that the biological methylation of amide bonds, as opposed to amino acids, was discounted. We propose a mechanism in which nature has evolved an elegant solution to this fascinating chemical puzzle. Harnessing the ability to methylate amide bonds of synthetic peptides at neutral pH in water to enhance their protease resistance and cell membrane permeability has significant applications in medicinal chemistry.

## METHODS

### Protein expression and purification

Insertion of Tobacco etch virus protease (TEV) cleavage sites after N-terminal his-tag and site-directed mutagenesis of OphA was carried out using a published protocol (*32*) with KOD Hot Start DNA Polymerase. All the proteins were overexpressed in *E. coli* BL21 (DE3) cells. All mutants were introduced into the OphAΔC6 construct. For protein expression, cells were grown at 37°C in Terrific Broth medium to OD_600_ (optical density at 600 nm) of around 1.0 and then cooled down to 16°C in ice-cold water bath (cold shock) before induction with 0.2 mM isopropyl-β-D-thiogalactopyranoside (IPTG) for 20 hours. For the expression of Y76F, cells were harvested after induction for 4 hours using 0.2 mM IPTG at 16°C due to cell death after overnight induction. For purification, cells were resuspended in lysis buffer [25 mM tris-HCl (pH 8.0), 500 mM NaCl, 10% glycerol, lysozyme (0.2 mg/ml; Sigma), DNase I (25 μg/ml; Sigma), and EDTA-free cocktail protease inhibitor (Roche Applied Science)] for 1 hour in a cold room. Cells were then lysed using a cell disrupter at 30 kpsi (Constant System Ltd.), and the cell lysate was cleared by centrifugation (40,000*g* for 30 min) at 4°C. The supernatant was loaded on a 20-ml nickel HisTrap FF column (GE Healthcare), washed with lysis buffer, and eluted with 250 mM imidazole. To remove imidazole and his-tag, eluents were dialyzed against 25 mM tris-HCl (pH 8.0), 10% glycerol, and 100 mM NaCl overnight in the presence of TEV protease at 4°C and loaded onto a second nickel column. The flow-through fractions were concentrated and subjected to size-exclusion chromatography (Superdex S200 or S75, GE Healthcare) preequilibrated with 20 mM tris-HCl (pH 8.0), 10% glycerol, and 100 mM NaCl. Fractions containing pure proteins were collected and concentrated to 64 mg/ml. Purification of OphAΔC6 and mutants followed the same procedure except for the absence of NaCl in all the purification buffers. EDTA (2 mM) was added to the flow-through fractions of the second nickel column to avoid precipitation during concentration. Concentrated protein was applied onto a gel filtration column, and fractions corresponding to the dimer were collected, concentrated to 30 to 60 mg/ml, and stored in 20 mM tris-HCl (pH 8.0), 10% glycerol.

For the expression of selenomethionine-substituted OphAΔC18 (Se-OphAΔC18), 50 ml of *E. coli* BL21 (DE3) cells was grown overnight in LB medium, spun down at 2500*g* for 10 min at room temperature, washed twice with phosphate-buffered saline, and transferred into 1 liter of M9 medium containing kanamycin (50 μg/ml). Cells were then grown at 37°C to an OD_600_ of 0.8, before the addition of lysine, threonine, and phenylalanine at a final concentration of 100 mg/liter and of leucine, isoleucine, and valine at a final concentration of 50 mg/liter. Protein expression was induced with 0.2 mM IPTG and 40 mg/liter of selenomethionine and expressed at 18°C for 20 hours. Protein was purified using the same methods as previously described except for the presence of 5 mM β-mercaptoethanol in all the purification buffers. After the gel filtration column, the protein was stored in 20 mM tris-HCl (pH 8), 10% glycerol, and 1 mM tris(2-carboxyethyl)phosphine (TCEP). The incorporation of selenium was confirmed by electrospray ionization–time-of-flight (ESI-TOF) MS.

To obtain OphAΔC6 with limited in vivo methylation (OphAΔC6-2h), cells were grown to an OD_600_ of 1.2 and then cold-shocked and induced with 0.4 mM IPTG for 2 hours before harvesting. Protein was purified as before and concentrated to 11.6 mg/ml and stored in buffer containing 25 mM tris-HCl (pH 8.0), 10% glycerol, and 2 mM TCEP.

### In vitro assay of OphAΔC6 and its mutants

To test the in vitro activity, OphAΔC6-2h and its mutants R72A, R72K, Y76F, Y98A, and Q172A were incubated at a concentration of 4 mg/ml with 5 mM SAM in 50 mM tris-HCl (pH 8.0). The extent of methylation at different time points was analyzed using trypsin digestion and tandem MS (MS/MS) (Fig. 3, A and B, and fig. S2A).

### In vitro guanidine rescue assay of OphAΔC6-R72A

To determine whether guanidine can rescue the activity of R72A, OphAΔC6-R72A was incubated with 5 mM SAM in the presence of 200 mM guanidine in 50 mM tris (pH 8.0). Methylation was then determined by trypsin digestion and MS/MS (Fig. 3B and fig. S6).

### pH profile assay of OphAΔC6-2h

To determine the pH rate profile of OphAΔC6, OphAΔC6-2h (4 mg/ml) was incubated with 5 mM SAM in appropriate buffers with a pH range from 4 to 10 (pH 4.0 to 5.0, 50 mM sodium citrate; pH 6.0 to 7.5, 50 mM bis-tris; pH 7.5 to 9.0, 50 mM tris; pH 9.0 to 10, 50 mM CHES). OphA precipitated at pH 4.0 and 5.0 sodium citrate buffer immediately, and reaction solution turned turbid after a 15-hour incubation at pH 6.0 bis-tris and pH 7.0 tris-HCl. The reactions were quenched after 15 hours by adding an equivalent volume of 8 M urea and further incubated for 1 hour. To make sure 4 M urea quenches the reaction, native OphAΔC6-2h was treated with 4 M urea and 5 mM SAM in tris-HCl for 1 hour. The samples were then diluted four times into ammonium bicarbonate (pH 8.5) and digested overnight with trypsin at 37°C (fig. S2B).

### Solvent kinetic isotope effect

D_2_O of 99.9% isotope purity from Sigma-Aldrich was used to prepare buffers and SAM stocks in D_2_O. To prepare OphAΔC6-2h in D_2_O, half of the OphAΔC6-2h proteins concentrated from gel filtration fractions were extensively buffer-exchanged into 25 mM tris buffer prepared in D_2_O (pD 8.0, where pD = pH* + 0.4, and pH* is the reading number on the pH meter) containing 10% glycerol at room temperature. The other half of OphAΔC6-2h proteins were stored in 25 mM tris (pH 8.0) containing 10% glycerol. Both proteins in H_2_O and D_2_O were aliquoted and frozen at −80°C for later experiments. To determine the solvent kinetic isotope effect, parallel reactions containing OphAΔC6-2h (4 mg/ml) and 5 mM SAM were carried out in 50 mM tris buffer (pH 8.0) or in 50 mM tris buffer in D_2_O (pD 8.0) for 7 hours before being quenched in 4 M urea. To determine the solvent viscosity effect on the reaction, another set of parallel reactions using a different batch of protein was performed in 50 mM tris-HCl (pH 8.0) containing varied concentrations of glycerol (0 and 9%) as the viscosigen. Glycerol (9%) has the same solvent viscosity as D_2_O. Methylation of individual reactions was analyzed similarly.

### ^18^O incorporation experiments

OphAΔC6-2h was buffer-exchanged into 25 mM tris (pH 8.0) in H_2_^18^O several times to the desired concentration (H_2_^18^O content was estimated to be 95%). Reactions were performed with SAM (at 4 mg/ml and 5 mM) for 24 hours at room temperature before freezing. The resulting products were examined by ESI-TOF MS. The results were identical to those in normal H_2_O.

### Ligand identification (SAM, SAH, and sinefungin) in OphA variants

OphA variants (OphAΔC18, OphAΔC6, Y63F, Y66F, R72A, Y76F, Y98A, and W400A) were quenched with trichloroacetic acid (TCA). Precipitation was removed before the supernatant was injected into HPLC-MS. To test whether SAH can be exchanged with SAM or sinefungin, OphAΔC6, Y63F, Y66F, R72A, Y76F, Y98A, and W400A at a concentration of about 30 to 40 mg/ml were incubated with 6 mM SAM or 10 mM sinefungin overnight before extensive dialysis or diafiltration. Y63F at a concentration of 30 mg/ml was also incubated with 50 mM sinefungin before extensive diafiltration. Around 50 crystals grown from conditions containing Y63F (30 mg/ml) and 50 mM sinefungin were fished, washed twice in its mother liquor, and then transferred into a 1.5-ml tube before quenched with TCA. All the quenched samples were analyzed with an analytical C18 reversed-phase column on HPLC-MS from Agilent.

### ESI-TOF MS and intact mass measurement

The protein sample (20 μl at 10 pM/μl) was desalted on-line through a Waters MassPREP column (2.1 mm × 10 mm), eluted with a gradient from solvent A [2% CH_3_CN, 97% H_2_O, 1% formic acid (FA)] to solvent B (97% CH_3_CN, 2% H_2_O, 1% FA), and delivered to a Waters LCT electrospray ionization mass spectrometer precalibrated using myoglobin. An envelope of multiple charged signals was obtained in positive ionization from 500 to 2000 mass/charge ratio (*m/z*) and deconvoluted using MaxEnt 1 software over the range of 44–48 kDa to 1 Da resolution to give the molecular mass of the protein.

### nLC-ESI-MS/MS analysis

#### Method 1

Samples were diluted to 10 μM (10 μl) with 25 mM ammonium bicarbonate and were digested at 37°C overnight using sequencing-grade trypsin (Promega) at 1:50. One picomole of the resultant peptides was aliquoted for MS analysis. Nanoliquid chromatography (nLC)–MS/MS data were recorded on a Sciex 5600+ mass spectrometer equipped with the Eksigent 2D Ultra NanoLC system using a Thermo Scientific Pepmap C18 column (75 μm × 15 cm) and trap (2 cm) in trap-elute configuration. The sample was held on the trap and washed for 5 min with loading buffer A (2% CH_3_CN, 98% H_2_O, 0.05% trifluoroacetic acid). The trap was switched in line with the column, and elution was performed with a linear gradient using eluent A (2% CH_3_CN, 98% H_2_O, 0.1% FA) and increasing eluent B (98% CH_3_CN, 2% H_2_O, 0.1% FA). The gradient was 95% A/5% B (at 0 min) linear to 60% A/40% B (at 6 min), linear to 5% A/95% B (at 9 min), held at 5% A/95% B until 12 min, linear to 95% A/5% B (at 14 min), and held at 95% A/5% B for 14 to 20 min. Mass spectra were acquired in positive-ion mode in information-dependent acquisition mode by performing 150 ms of MS followed by 80 ms of MS/MS analysis of the 10 most intense peaks seen by MS. These masses were then excluded from analysis for the next 10 s. MS spectra were acquired from 400 to 1350 *m/z*, and MS/MS spectra were acquired from 95 to 2000 *m/z*. MS/MS data were extracted using the SCIEX PeakView software and analyzed using the Mascot 2.6 search engine (Matrix Science) against a sequence database containing OphA (and mutants). The data were searched with tolerances of 20 ppm for the precursor and 0.1 Da for fragment ions, trypsin as the cleavage enzyme, and three missed cleavages. A methyl modification (+14.01565 atomic mass unit monoisotopic) was added to the modification database for V, G, I, and A and used as a variable modification. The potential methylation sites were further verified by manual interpretation of subsequent analysis under the same conditions as above but utilizing product ion mode scanned specifically for the masses of interest of the C-terminal trypsin-digested peptide (and mutants) in the various methylated states. The MS spectra were used to create extracted ion chromatograms of the masses of interest ±0.05 *m/z*, and the peak areas were measured using the PeakView software.

#### Method 2

In vitro samples (10 μl) were transferred into 1.7-ml Protein LoBind tubes (Eppendorf). After denaturing for 1 hour using 6 μl of 8 M urea, a final concentration of 0.5 M ammonium bicarbonate, 2 mM TCEP (pH 8.0), and Trypsin Gold (Promega) in a molar ratio of 1:50 was added to the sample and incubated overnight at 37°C. The samples were then desalted and purified using C18 ZipTips (Millipore) and eluted with 90% acetonitrile (ACN), 0.1% FA. After drying the samples in a SpeedVac (Eppendorf), peptides were resuspended in 15 μl of 20% ACN, 0.1% FA, and transferred to glass vials for MS analysis. An injection volume of 4 μl was used for MS samples. HPLC-MS/MS data were recorded on a Thermo Scientific Fusion mass spectrometer equipped with a Dionex Ultimate 3000 UHPLC system using a nLC column (200 mm × 75 μm) packed using Vydac 5-μm particles with a 300 Å pore size (Hichrom Limited). A 75-μm emitter with a 10-μm tip was used for electrospray (New Objective). Elution was performed with a linear gradient using water with 0.1% (v/v) FA (solvent A) and ACN with 0.1% (v/v) FA (solvent B) at a flow rate of 0.3 μl/min. The column was equilibrated with 20% solvent B for 5 min, which was followed by a linear increase of solvent B to 85% over 32 min and a final elution step with 85% solvent B for 2 min. Mass spectra were acquired in positive-ion mode with the following settings: spray voltage at 2200 V and an S-lens level at 60. Full MS was done at a resolution of 60,000 [automatic gain control (AGC) target, 4 × 10^5^; maximum ion trap (IT), 50 ms; range, 300 to 1800 *m/z*], and data-dependent MS/MS was performed at a resolution of 15,000 (AGC target, 5 × 10^5^; maximum IT, 500 ms; isolation window, 2.2) using higher-energy collisional dissociation (HCD) with a stepped normalized collision energy of 14, 18, and 22. The inclusion list contained masses of the trypsinized fragments encoding the omphalotin peptide (or one of the variants) and its different methylation states. Data were processed using Thermo Fisher Xcalibur software and MaxQuant, as previously described (*12*).

### Crystallization

All crystals were obtained using freshly prepared proteins from the dimer fractions of gel filtration chromatography. To obtain complex crystals of OphAΔC18 or OphAΔC6, proteins were incubated with 3 mM SAM or SAH prior to crystallization. Hanging drop vapor diffusion method was used for all crystals unless otherwise specified.

Crystals of “apo” OphAΔC18 (in reality, predominantly bound with SAM) and OphAΔC18 in complex with SAM were obtained at 16°C at a concentration of 40 to 50 mg/ml in the condition containing 0.06 M NaBr, 2.67% 1,6-hexanediol, 0.1 M MES (pH 6.0), 14% polyethylene glycol (PEG) 6000. Crystals appeared within 1 week, continued to grow within 2 weeks, and were then transferred to the reservoir solution supplemented with 25% glycerol and 5% PEG 6000 before flash freezing in a nitrogen stream. The complex of OphAΔC18 (50 mg/ml) with SAH was crystallized using sitting drop method in a condition containing 0.04 M ammonium citrate tribasic, 0.1 M sodium citrate (pH 5.5), and 27% PEG monomethyl ether (MME) 5000. Crystals grew to full size within 2 weeks and were then transferred to their mother liquor supplemented with 10% PEG MME 5000 before flash freezing. The Se-OphAΔC18 (30 mg/ml) SAM complex was crystallized in a condition containing 0.2 M NaCl, 0.1 M bis-tris (pH 6.0), and 20% PEG 3350. Crystals appeared within 2 days and matured within 2 weeks. These crystals were flash-frozen in mother liquor supplemented with 20% PEG 3350.

Complex crystals of OphAΔC6 (30 mg/ml) with SAM or SAH, or mutant Y65F (30 mg/ml) with SAM, were obtained in a condition containing 0.02% octyl β-d-glucopyranoside, 0.2 M KCl, 0.1 M bis-tris (pH 6.5), and 20% PEG 4000. Crystals were transferred in the reservoir solution supplemented with 20% PEG 4000 and flash-frozen in liquid nitrogen.

For crystallization of OphAΔC6 mutants (Y63F, Y66F, Y76F, Y98A, R72A, and W400A in complex with SAM, and Y76F and R72A in complex with SAH), 1 μl of protein at a concentration of 30 to 50 mg/ml was mixed with 1 μl of reservoir solution [0.3 to 0.4 M KSCN, 1.5 to 1.9 M sodium malonate, and 0.1 M bicine (pH 9.0)]. Two shapes of crystals, brick-shaped and octahedral-shaped, appeared for Y63F, Y66F, R72A, and Y98A with SAM, while only brick-shaped crystals were observed for Y76F with SAM or SAH and for R72A with SAH. All crystals were flash-frozen in their mother liquor supplemented with 0.7 to 0.9 M sodium malonate.

For crystallization of OphAΔC6 mutants (W400A and Y63F) in complex with sinefungin, protein (20 to 30 mg/ml) was incubated with 10 mM sinefungin overnight at 4°C before crystallization. Brick-shaped crystals appeared within 2 days and matured within 2 weeks in the condition containing 0.3 to 0.4 M KSCN, 0.1 M bicine (pH 8.0 for Y63F and pH 8.5 for W400A), and 1.3 to 1.5 M sodium malonate. Crystals were then soaked in 10 mM sinefungin for 2 days and then transferred to a cryoprotectant (mother liquor supplemented with 1.4 to 1.7 M sodium malonate and 2 mM sinefungin) before flash freezing in liquid nitrogen. To increase the occupancy of sinefungin in the Y63F mutant, protein (30 mg/ml) was incubated with 50 mM sinefungin for 3 days before crystallization. After 1 week, crystals were soaked in 70 mM sinefungin overnight before flash freezing in liquid nitrogen.

### Data collection, reduction, and refinement

X-ray diffraction data were recorded at either Diamond Light Source beamline I04-1 (λ = 0.9281 Å), I03 (λ = 0.9763 Å), or I24 or collected in-house, on a Rigaku 007HFM copper (λ = 1.54 Å) rotating anode x-ray generator with a Saturn 944 charge-coupled device (CCD) detector at 100 K. Data were reduced, integrated, and scaled using a toolbox autoPROC (*33*–*35*), xia2 (*36*–*38*), or HKL2000(*39*); OphAΔC6 Y76F-SAM complex data were reindexed to P2_1_2_1_2 in CCP4(*40*) and further merged with Aimless in CCP4 (*40*). Automated structure solution pipeline CRANK2 (*41*) was used for experimental phasing using selenium single-wavelength anomalous diffraction (SAD) methods for the Se-OphAΔC18 data set. The model was further improved and refined using COOT (*42*), REFMAC5 (*43*), and Phenix (*44*). All the other structures were solved by molecular replacement with OphAΔC18 as the initial search model using Phaser (*45*) and refined as described above. Each final model was verified with MolProbity (*46*). Refinement statistics were summarized in table S1, and electron density in fig. S6. Although the main chain electron density of the substrate peptide was clear, despite the relatively high resolution of the structures, the side chains of the substrate peptides could not be interpreted unambiguously in most cases. We attribute this to disorder within the register, that is, substrate peptide docks in multiple conformations in the crystal, which, although sharing the same backbone orientation, have different side chains. Consequently, although we have modeled a particular register in the structures, our interpretations have avoided placing weight on the identity of the side chain at position i. To obtain the ratio of SAM, SAH, or sinefungin in crystals, structures of Y63F-sinefungin (50 mM), R72A-SAM, Y76F-SAM, Y98A-SAM, and W400A-sinefungin were refined for many rounds by Phenix until occupancy of SAH, SAM, or sinefungin (starting point is 0.5 occupancy of SAM/sinefungin and SAH) no longer change. Electrostatic surfaces were calculated with CCP4MG (*47*). All crystallographic figures were generated using Pymol (Schrödinger LLC). Coordinates and data have been deposited with the wwPDB (Worldwide Protein Data Bank).

### MD simulations and QM calculations

All MD simulations were performed under isothermic-isobaric conditions at 298 K using the GROMOS package of programs (*48*). The atomistic GROMOS force field 54A7 (*49*) was used with the simple point charge (SPC) model as water model. Initial parameters for SAM and SAH were taken from the Automated Topology Builder server (*50*) and adjusted to match the parameters of related substructures in amino acids. The bond lengths were constrained to the ideal values applying the SHAKE algorithm (*51*). The temperature was maintained close to its reference value *T* = 298 K by weak coupling to a temperature bath with a relaxation time of 0.1 ps (*52*). The pressure was maintained close to its reference value *P* = 1.013 bar (1 atm) by weak coupling to a pressure bath with a relaxation time of 0.5 ps and using the isothermal compressibility *k*_T_ = 4.575 × 10^−4^ (kJ mol^−1^ nm^−3^)^−1^. Newton’s equations of motion were integrated using the leap-frog scheme (*53*) with a time step of 2 fs. A reaction field force (*54*) was applied using the relative dielectric permittivity ε_rf_ = 61(*55*). As initial coordinates, the crystal structure of OphAΔC6 with SAM was taken. All crystal waters were removed with the exception of one water molecule buried in each binding pocket. The two magnesium cations were included. The missing loop between residues 268 and 277 in monomer A was modeled using the webserver ModLoop (*56*). The missing loops between residues 380 and 405 in monomer A and between residues 379 and 404 in monomer B were not modeled due to their length, that is, the substrates in the binding pockets were not connected to the parent proteins. The dimer complex was energy-minimized in vacuum and solvated in a cubic box of 21516 SPC water molecules. The solvent was energy-minimized with the protein position restrained. A thermalization in five steps from 60 to 298 K was performed with the position restraints decreased from 2.5 × 10^4^ kJ mol^−1^ nm^−2^ to zero, with the first four steps being 20 ps under constant-volume condition and the last step being 500 ps under isothermic-isobaric condition. A production run of 20 ns was performed. The simulations were analyzed in terms of backbone atom-positional rmsd and secondary structure motifs using the GROMOS++ analysis programs (fig. S8) (*57*). As QM calculations require a defined location of the proton in the product state for convergence, a snapshot where the water molecules formed a hydrogen-bonding network up to the deprotonated carboxy group of SAM was taken from the MD simulation as a starting point. If necessary, the orientation of key residues in the binding pocket was modified using PyMol (Schrödinger LLC) to obtain a hydrogen-bonding network similar to that observed in the crystal structure of OphAΔC6. The final geometry is shown in Fig. 5B.

All electronic structure calculations were performed with the quantum chemistry software package Gaussian09 Rev. D1 (Gaussian Inc.), using Kohn-Sham density functional theory (*58*). B3LYP (*59*, *60*) and TPSSH (*61*) were used as exchange correlation functional. The orbitals were expanded in the 6-31G** basis set (*62*, *63*) in conjunction with density fitting for the two-electron integrals (*64*, *65*). The size of the integration grid was chosen as UltraFine. To account for solvation effects, a reaction field within the integral equation formalism model (*66*) was applied. As solvent, diethylether with a dielectric constant of ε = 4.24 was chosen, which is a common approximation for the surrounding enzyme environment (*67*). For the minimization of the electronic energy with respect to the nuclear coordinates and the location of the transition state, atoms far away from the reaction center were kept frozen. More specifically, the methyl group of SAM was chosen as center, and all atoms with a distance >7 Å to it were constrained (fig. S8). Once a saddle point was located, a unique and coordinate-independent reaction coordinate, the so-called intrinsic reaction coordinate (IRC), was followed. By definition, this corresponds to an imaginary minimum energy trajectory in mass-weighted Cartesian coordinates with the initial direction indicated by the normal mode of the imaginary frequency of the transition state (*68*). In essence, this trajectory corresponds to the steepest decent path or minimum energy path (*69*, *70*). The IRCs of step 1 and 2 are shown in fig. S8.

## Supplementary Material

http://advances.sciencemag.org/cgi/content/full/4/8/eaat2720/DC1

## SUPPLEMENTARY MATERIALS

Supplementary material for this article is available at http://advances.sciencemag.org/cgi/content/full/4/8/eaat2720/DC1 Fig. S1. Structural analysis of OphA. Fig. S2. pH rate profile of OphAΔC6-2h at different pH values (7.0 to 10.0). Fig. S3. Experimental data for complexes. Fig. S4. Superimposition of OphAΔC6 structures. Fig. S5. Sequence alignment of the methyltransferase domain of OphA (Ompol1_2087). Fig. S6. Mass analysis of all mutants discussed in the manuscript. Fig. S7. All in vivo inactive mutants have similar inactive confirmations. Fig. S8. MD simulations and QM calculations. Fig. S9. Kinetic isotope effect and solvent viscosity effect studies of OphAΔC6-2h. Fig. S10. Alternate register for substrate peptide. Table S1. Crystallographic data. Table S2. Occupancy of SAM/SFG and SAH in OphA variants. References (*71*–*75*)
