# Supplementary material for: A molecular mechanism for the enzymatic methylation of nitrogen atoms within peptide bonds
Source: Sci Adv. 2018 Aug 24;4(8):eaat2720. doi: 10.1126/sciadv.aat2720 (PMC6108569; doi:10.1126/sciadv.aat2720)
Supplement: http://advances.sciencemag.org/cgi/content/full/4/8/eaat2720/DC1 [file supp_4_8_eaat2720__index.html]

Science Advances | Science Advances

## Supplementary Materials

**This PDF file includes:**

- Fig. S1. Structural analysis of OphA.
- Fig. S2. pH rate profile of OphAΔC6-2h at different pH values (7.0 to 10.0).
- Fig. S3. Experimental data for complexes.
- Fig. S4. Superimposition of OphAΔC6 structures.
- Fig. S5. Sequence alignment of the methyltransferase domain of OphA (Ompol1\_2087).
- Fig. S6. Mass analysis of all mutants discussed in the manuscript.
- Fig. S7. All in vivo inactive mutants have similar inactive confirmations.
- Fig. S8. MD simulations and QM calculations.
- Fig. S9. Kinetic isotope effect and solvent viscosity effect studies of OphAΔC6-2h.
- Fig. S10. Alternate register for substrate peptide.
- Table S1. Crystallographic data.
- Table S2. Occupancy of SAM/SFG and SAH in OphA variants.
- References (*71*–*75*)

Download PDF

**Files in this Data Supplement:**

- Adobe PDF - aat2720\_SM.pdf
