# Supplementary material for: A molecular mechanism for the enzymatic methylation of nitrogen atoms within peptide bonds
Source: Sci Adv. 2018 Aug 24;4(8):eaat2720. doi: 10.1126/sciadv.aat2720 (PMC6108569; doi:10.1126/sciadv.aat2720)
Supplement: http://advances.sciencemag.org/cgi/content/full/4/8/eaat2720/DC1 [file aat2720_SM.pdf]

Science Advances
AAAS

advances.sciencemag.org/cgi/content/full/4/8/eaat2720/DC1

Supplementary Materials for

A molecular mechanism for the enzymatic methylation of nitrogen atoms within peptide bonds

Haigang Song, Niels S. van der Velden, Sally L. Shiran, Patrick Bleiziffer, Christina Zach, Ramon Sieber, Aman S. Imani, Florian Krausbeck, Markus Aebi, Michael F. Freeman, Sereina Riniker*, Markus Künzler*, James H. Naismith*

*Corresponding author. Email: sriniker@ethz.ch (S.R.); mkuenzle@ethz.ch (M.K.); naismith@strubi.ox.ac.uk (J.H.N.)

Published 24 August 2018, *Sci. Adv.* **4**, eaat2720 (2018)
DOI: 10.1126/sciadv.aat2720

This PDF file includes:

Fig. S1. Structural analysis of OphA.
Fig. S2. pH rate profile of OphAΔC6-2h at different pH values (7.0 to 10.0).
Fig. S3. Experimental data for complexes.
Fig. S4. Superimposition of OphAΔC6 structures.
Fig. S5. Sequence alignment of the methyltransferase domain of OphA (Ompol1_2087).
Fig. S6. Mass analysis of all mutants discussed in the manuscript.
Fig. S7. All in vivo inactive mutants have similar inactive confirmations.
Fig. S8. MD simulations and QM calculations.
Fig. S9. Kinetic isotope effect and solvent viscosity effect studies of OphAΔC6-2h.
Fig. S10. Alternate register for substrate peptide.
Table S1. Crystallographic data.
Table S2. Occupancy of SAM/SFG and SAH in OphA variants.
References (*71–75*)

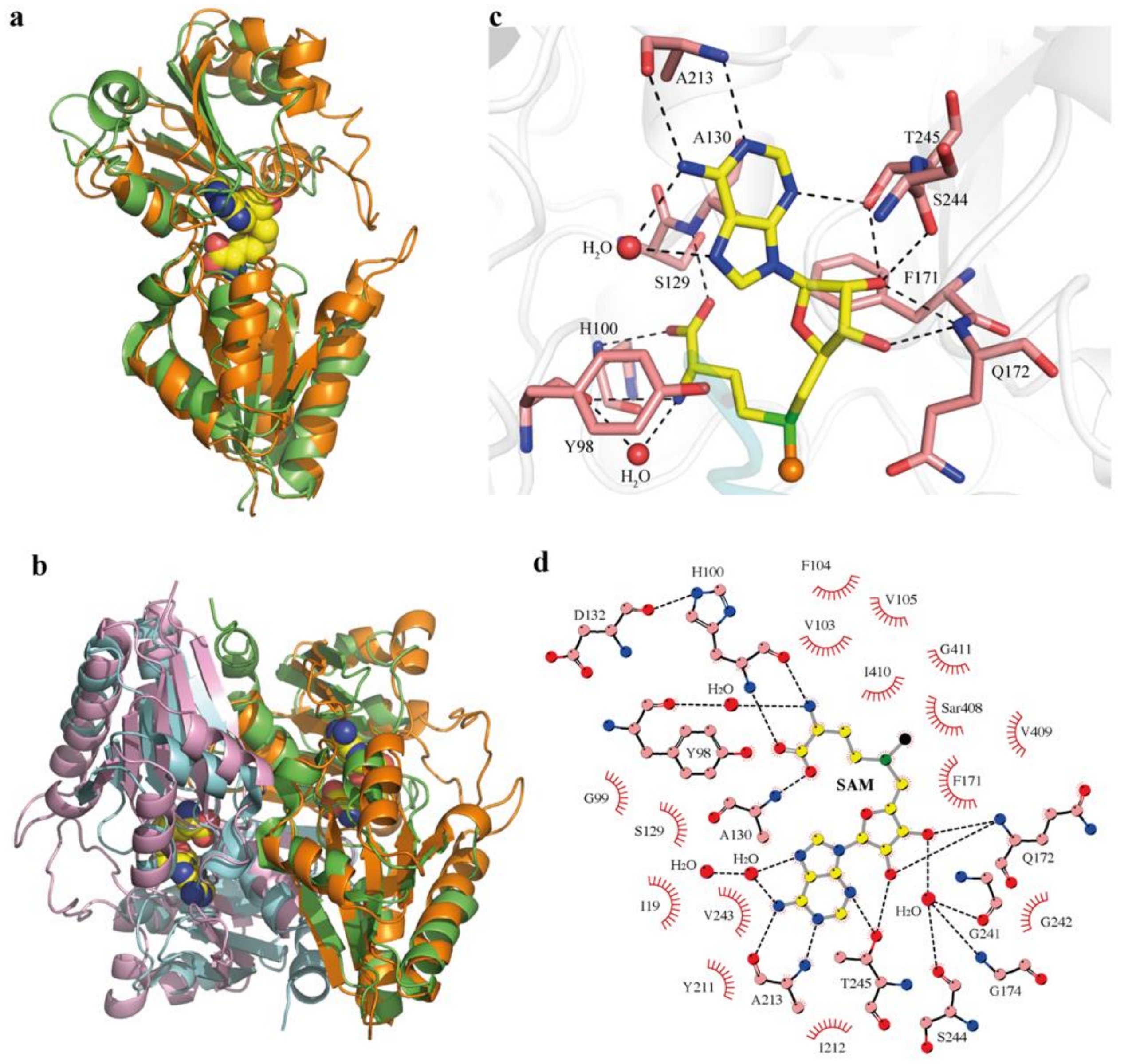

Fig. S1. Structural analysis of OphA. a The compact N-terminal domain of OphA (Thr 7 to Lys 251, colored orange) has two lobes that bind SAM /SAH (shown in spheres). The domain is related to other SAM dependent enzymes. The superposition of uroporphyrinogen III C methyltransferase (*71*) (PDB 1VE2) has a root mean square deviation 2.4 Å over 197 Cα atoms is shown in green. A molecule of SAH is shown as spheres. The additional loops of OphA that fold over the active site are visible. **b** Both OphA (orange and pale pink) and uroporphyrinogen III C methyltransferase (green and cyan) (*71*) (PDB 1VE2) adopt the same 'back to back dimer' arrangement. Only the N-terminal domain of OphA is shown for clarity. **c** SAM (and SAH) make a large number of highly specific interactions with the protein. **d**. Ligplot showing the interactions between SAM and protein.

a

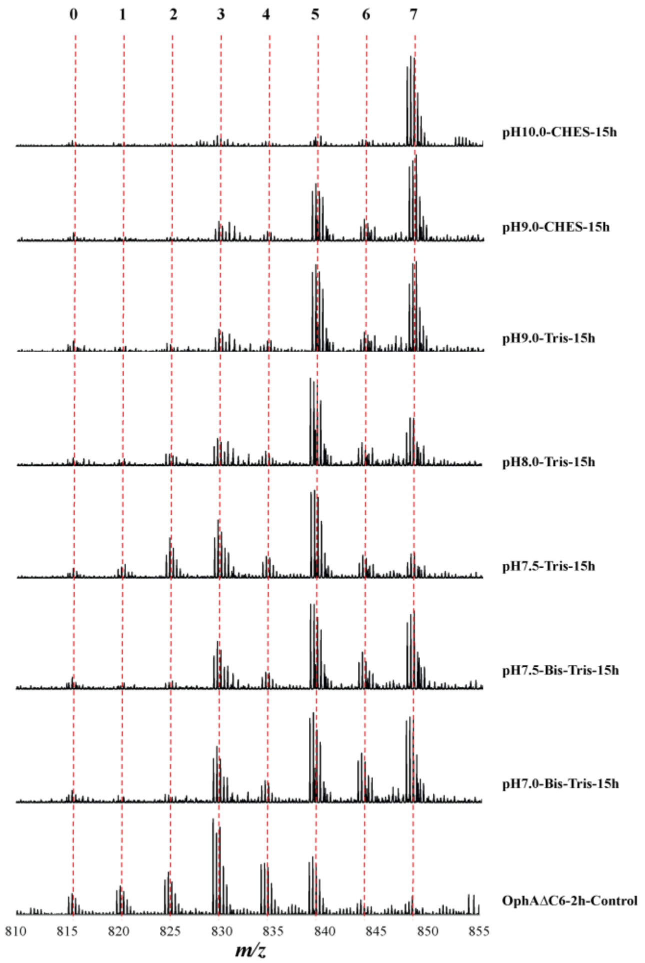
0
1
2
3
4
5
6
7
pH10.0-CHES-15h
pH9.0-CHES-15h
pH9.0-Tris-15h
pH8.0-Tris-15h
pH7.5-Tris-15h
pH7.5-Bis-Tris-15h
pH7.0-Bis-Tris-15h
OphAΔC6-2h-Control
810
815
820
825
830
835
840
845
850
855
m/z

b

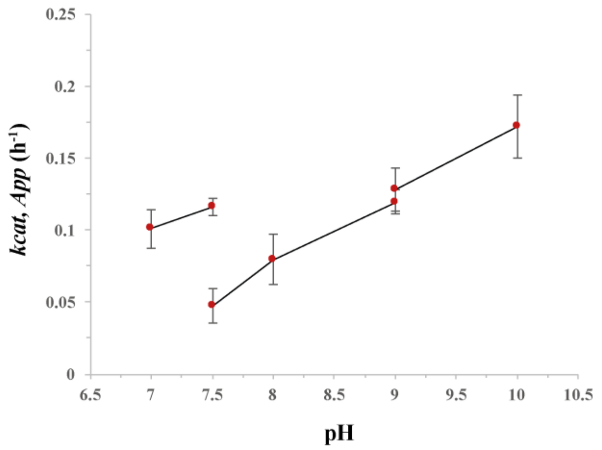
0.25
0.2
0.15
0.1
0.05
0
kcat, App (h-1)
6.5
7
7.5
8
8.5
9
9.5
10
10.5
pH

Fig. S2. pH rate profile of OphAΔC6-2h at different pH values (7.0 to 10.0). a MS analysis of OphAΔC6-2h incubated with SAM for 15 h at different pH. Reaction was quenched by further incubating in the presence of 4 M urea for 1h h before trypsin digestion. Control was done by incubating OphAΔC6-2h with SAM and 4 M urea overnight. Reactions for each pH were performed in triplicates (n=3) and only one representative in each pH was shown. **b** The average extent of methylation for OphA was calculated from integration the peaks of all eight species (species with 0 to 7 methylations). To estimate the k_{cat}, the change of average extent of methylation compared to control was calculated and divided by the time of SAM incubation. The error bars show standard deviation (n=3).

a

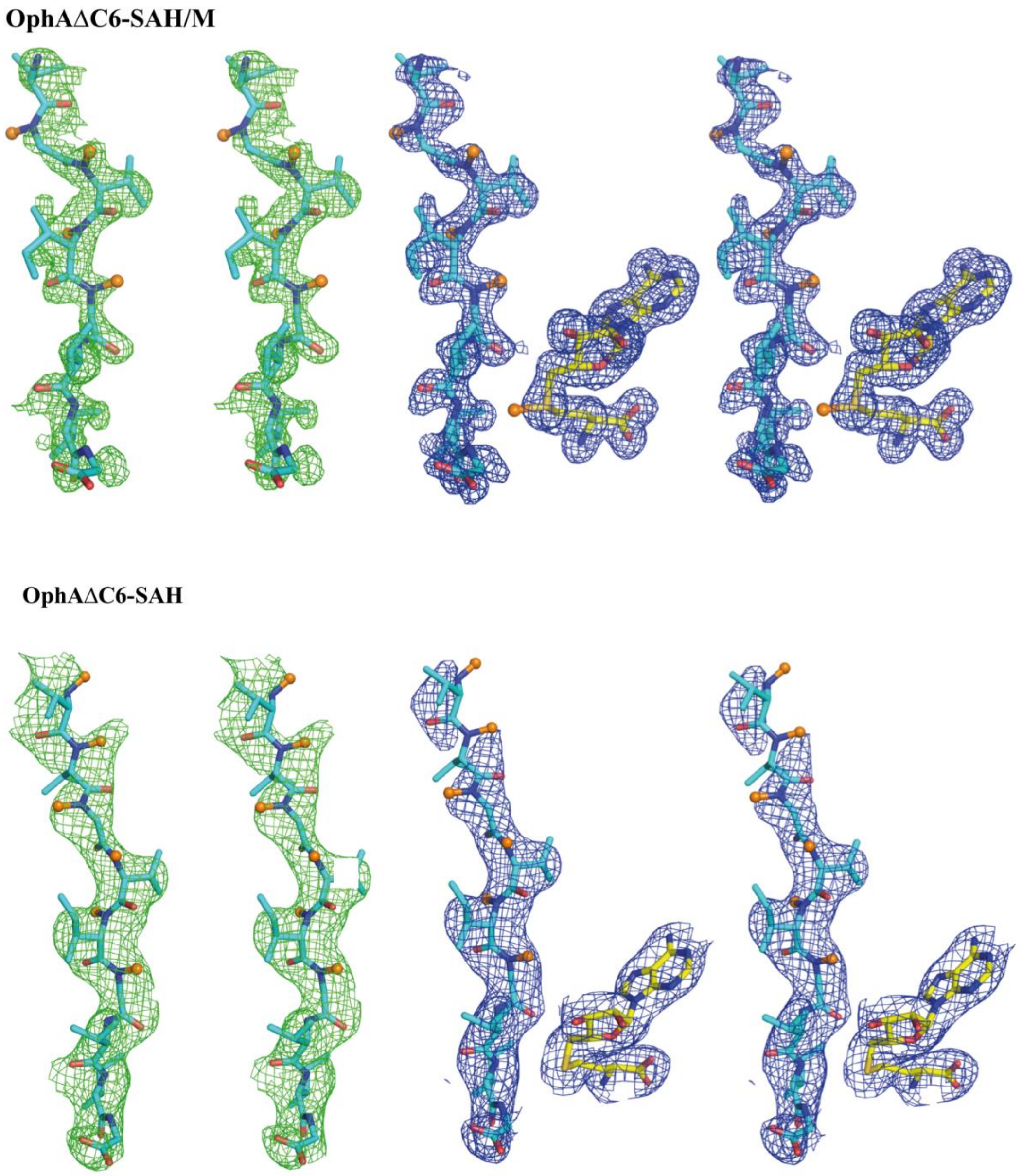

OphA-ΔC6-Y63F-SAH

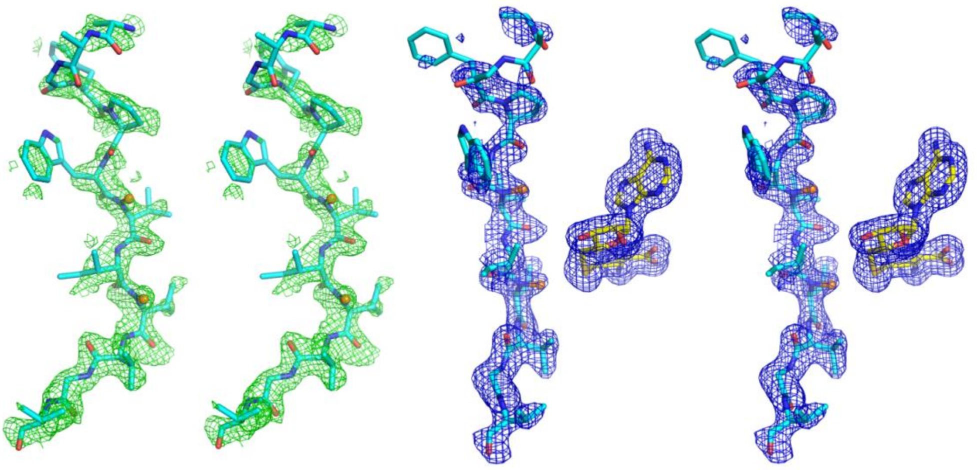

OphA-ΔC6-Y63F-sinefungin (50mM)

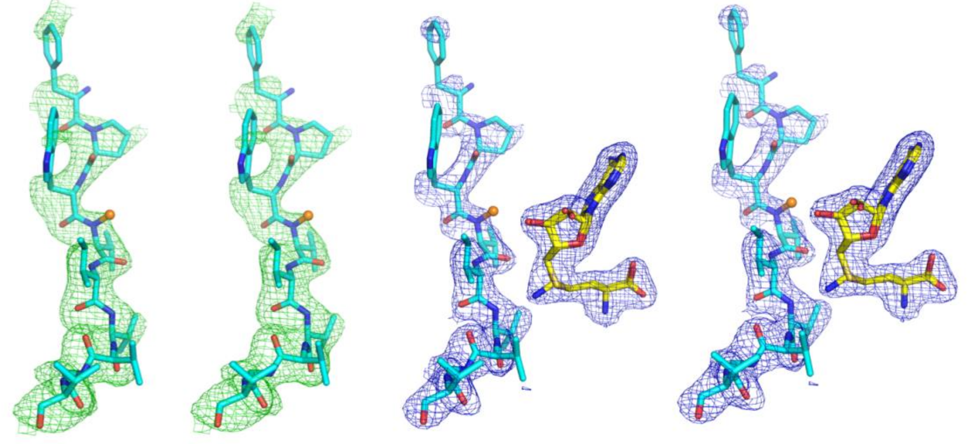

OphAΔC6-Y98A-SAM

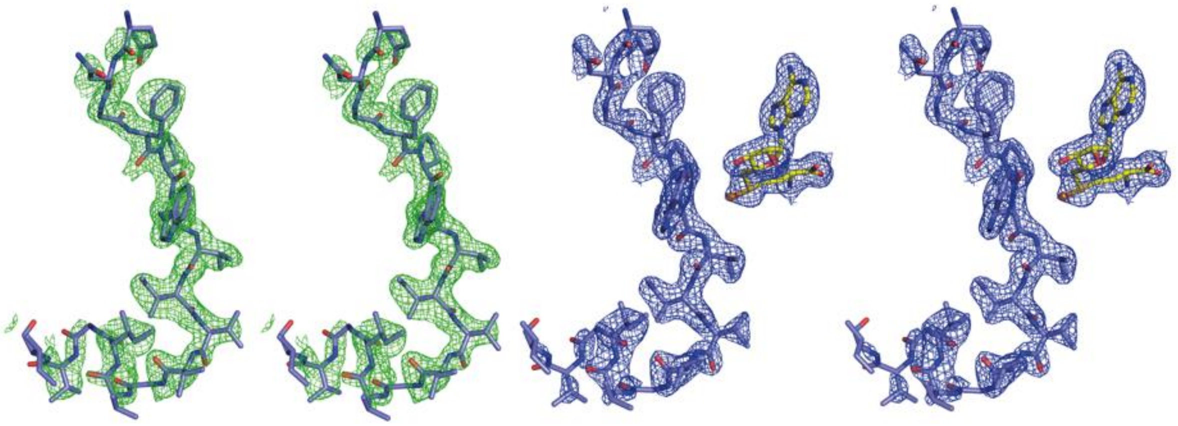

OphAΔC6-Y66F-SAM

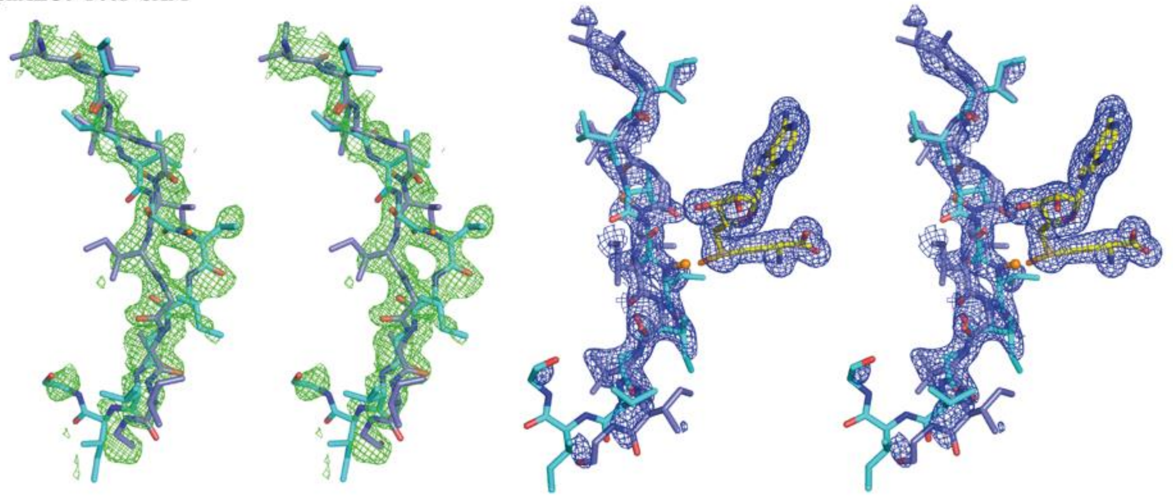

OphAΔC6-Y76F-SAM

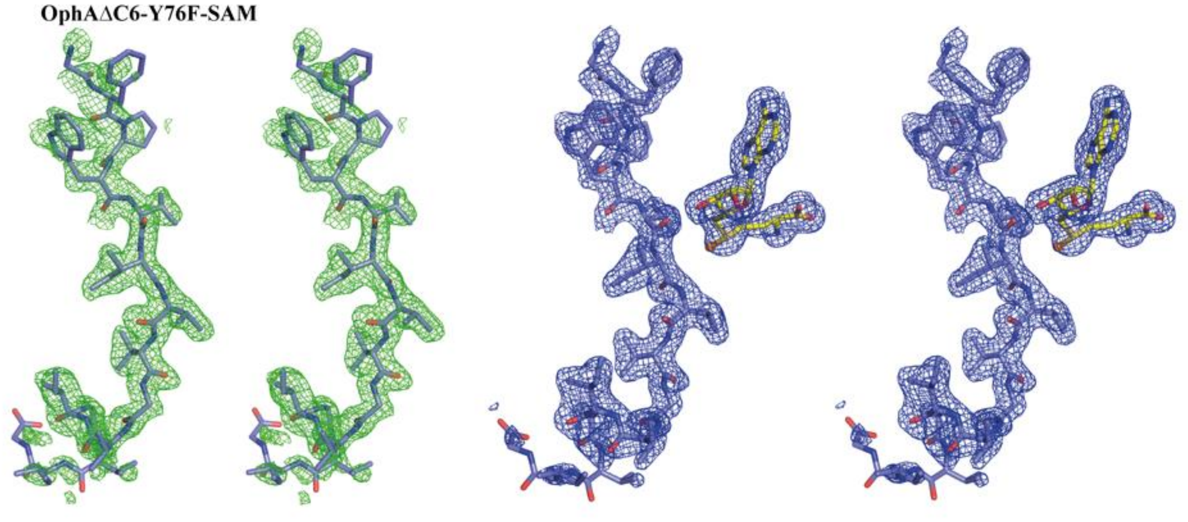

OphAΔC6-Y76F-SAH

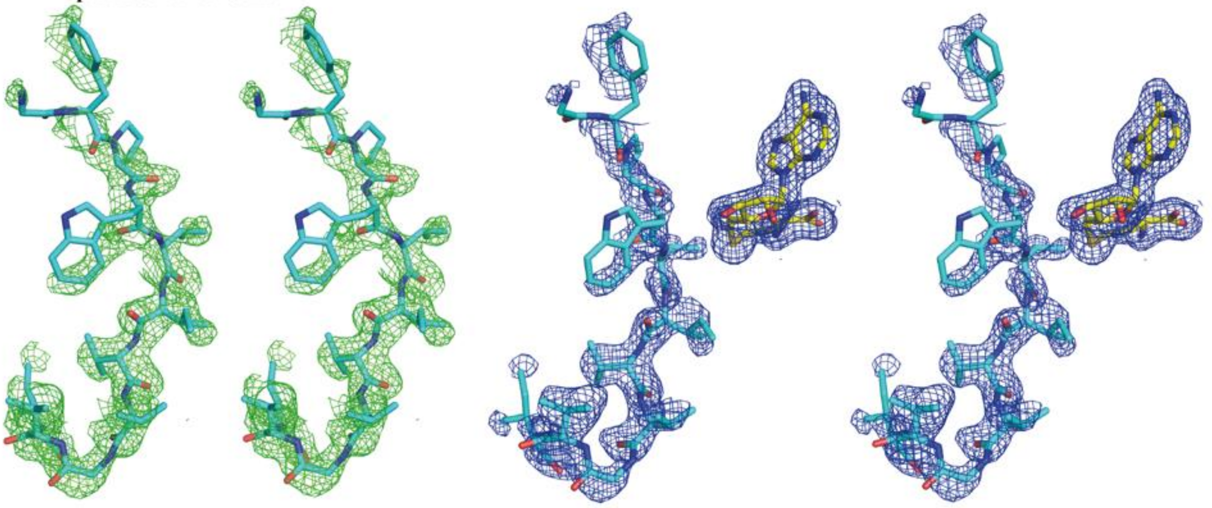

OphAΔC6-R72A-SAM

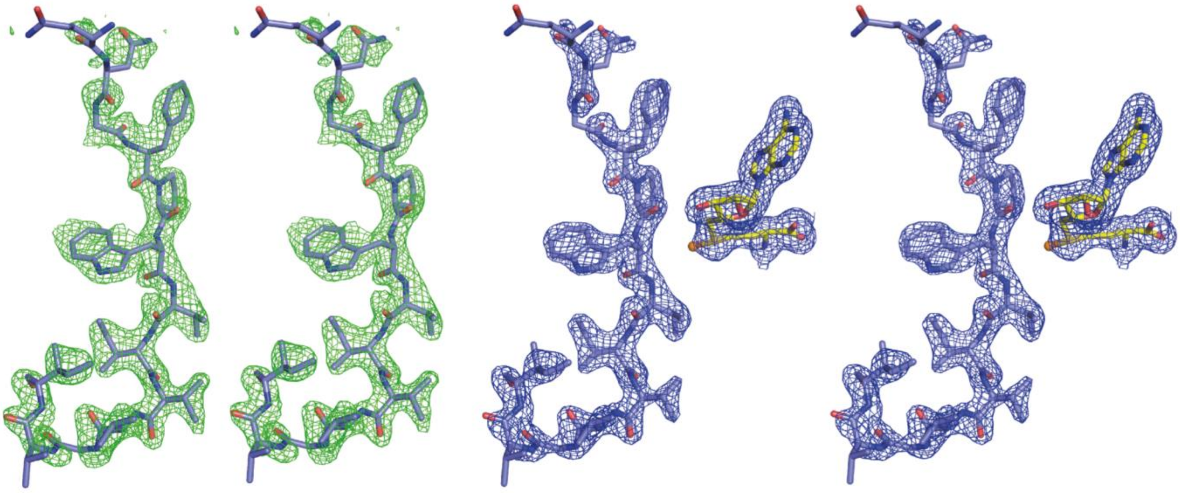

OphAΔC6-R72A-SAH

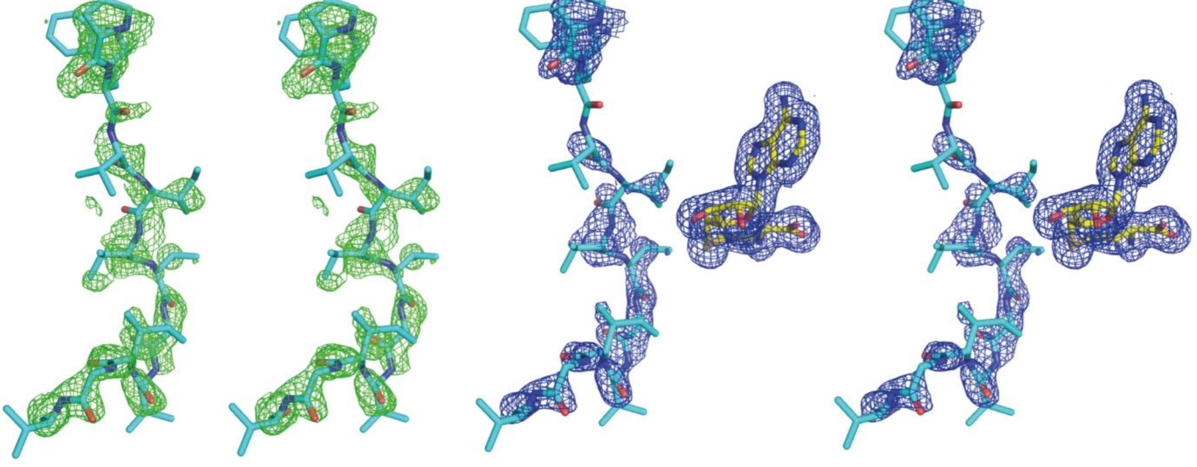

OphAΔC6-W400A-SAM

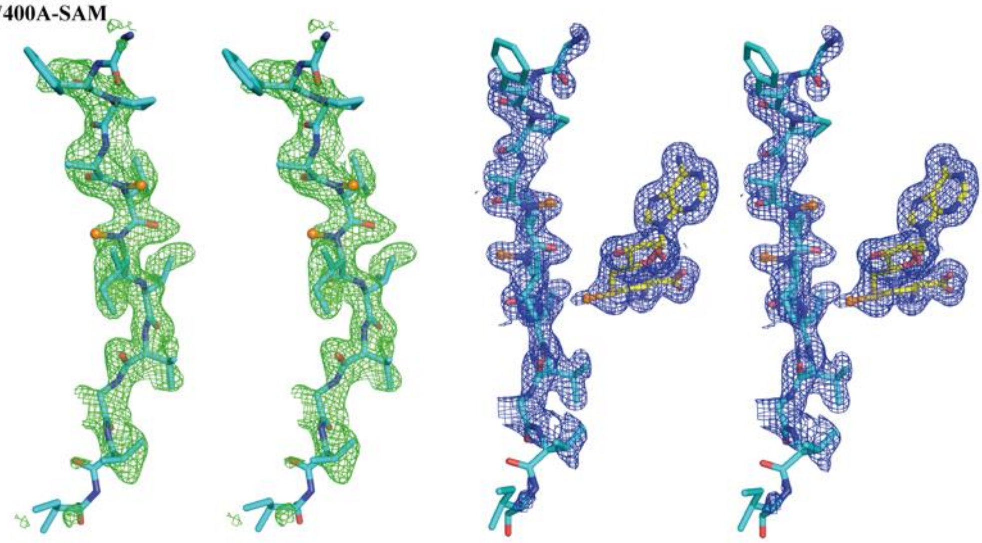

OphAΔC6-W400A-Sinefungin

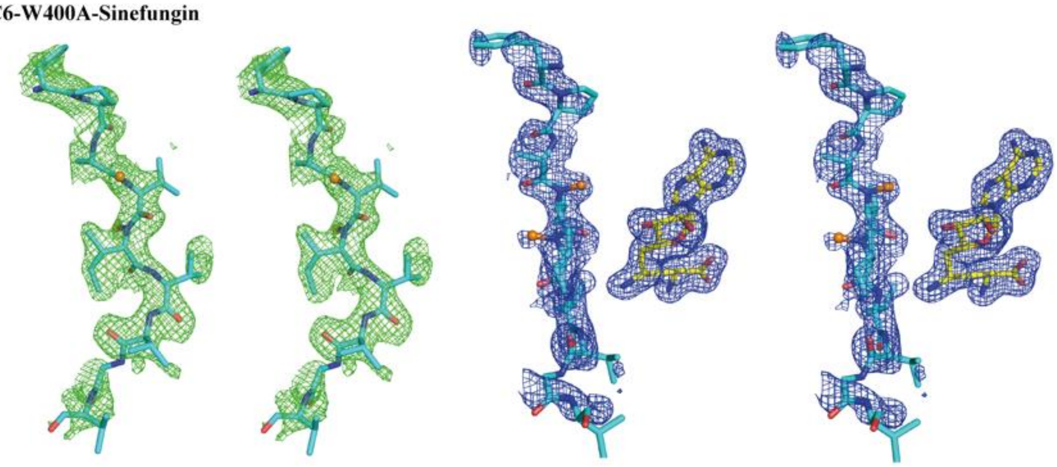

b

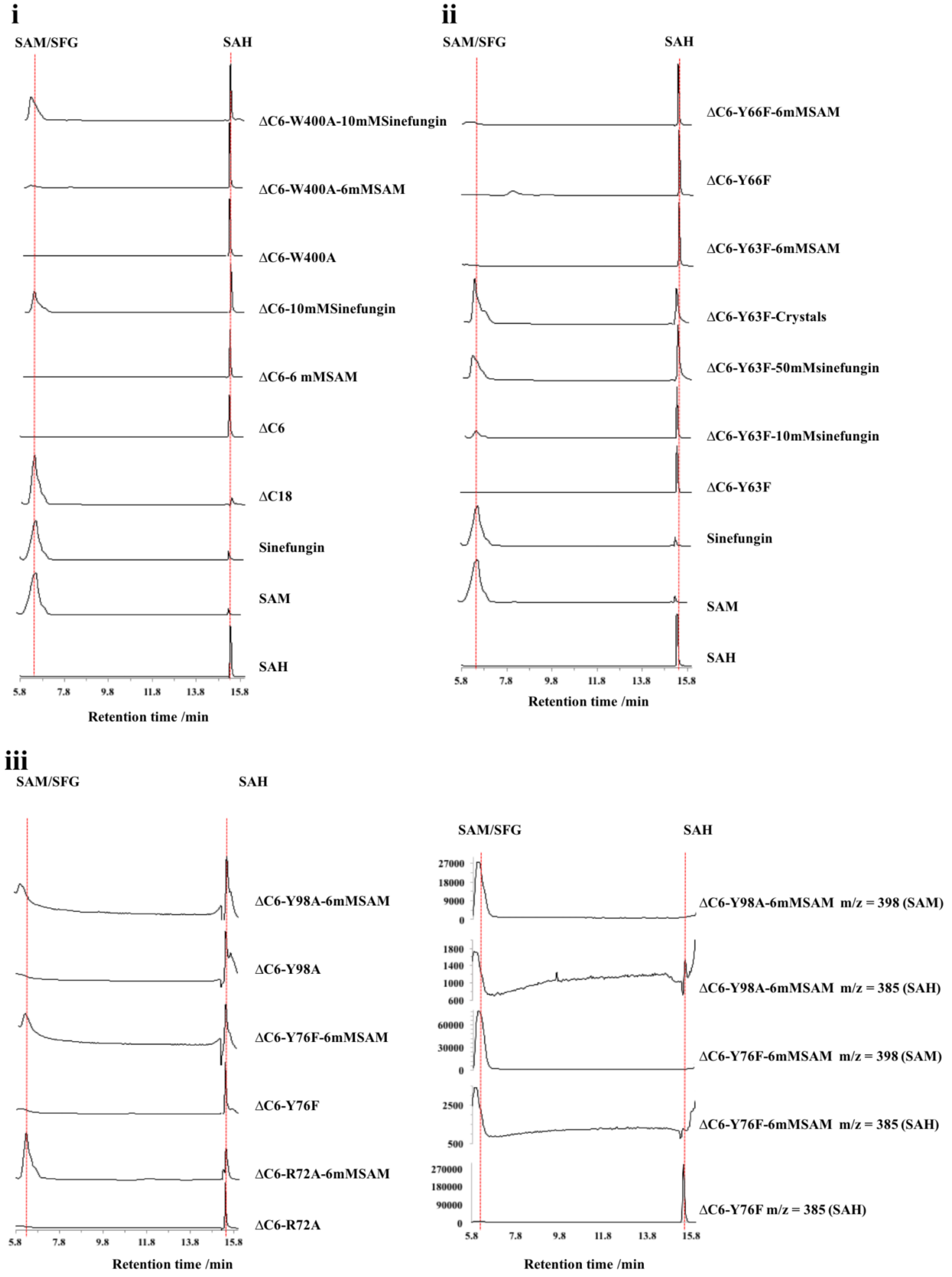
i
SAM/SFG
SAH
ΔC6-W400A-10mMSinefungin
ΔC6-W400A-6mMSAM
ΔC6-W400A
ΔC6-10mMSinefungin
ΔC6-6 mMSAM
ΔC6
ΔC18
Sinefungin
SAM
SAH
5.8 7.8 9.8 11.8 13.8 15.8
Retention time /min
ii
SAM/SFG
SAH
ΔC6-Y66F-6mMSAM
ΔC6-Y66F
ΔC6-Y63F-6mMSAM
ΔC6-Y63F-Crystals
ΔC6-Y63F-50mMsinefungin
ΔC6-Y63F-10mMsinefungin
ΔC6-Y63F
Sinefungin
SAM
SAH
5.8 7.8 9.8 11.8 13.8 15.8
Retention time /min
iii
SAM/SFG
SAH
ΔC6-Y98A-6mMSAM
ΔC6-Y98A
ΔC6-Y76F-6mMSAM
ΔC6-Y76F
ΔC6-R72A-6mMSAM
ΔC6-R72A
5.8 7.8 9.8 11.8 13.8 15.8
Retention time /min
SAM/SFG
SAH
27000 18000 9000 0
ΔC6-Y98A-6mMSAM m/z = 398 (SAM)
1800 1400 1000 600
ΔC6-Y98A-6mMSAM m/z = 385 (SAH)
60000 30000 0
ΔC6-Y76F-6mMSAM m/z = 398 (SAM)
2500 500
ΔC6-Y76F-6mMSAM m/z = 385 (SAH)
270000 180000 90000 0
ΔC6-Y76F m/z = 385 (SAH)
5.8 7.8 9.8 11.8 13.8 15.8
Retention time /min

Fig. S3. Experimental data for complexes. a. Wall eye stereo view of the simulated annealing Fo-Fc omit maps (2σ, green) and 2Fo-Fc maps (1σ, blue). The 2Fo-Fc maps (blue) are from the final refined structure. The final model is shown for clarity. As we point out in the main text the density for side chains of the substrate peptide in some complexes suggests the residue at position i varies within the crystal. Our interpretations do not rely on the register (the side chain of the residue) at position i. The methyl of SAM was modelled at partial occupancy in OphAΔC6-SAM structure. **b.** HPLC-MS analysis of OphA variants quenched by TCA**. i-ii,** HPLC analysis of ΔC6 variants. Proteins were quenched after purification or after incubation with SAM or sinefungin. Proteins were extensively dialyzed or diafiltration before quenching. The presenc of SAM, sinfungin and SAH were also confirmed by SIC chromatography at *m/z* of 399.4 (SAM), 385.4 (SAH) , and 382.4 (sinefungin) **iii.** HPLC-MS analysis of inactive mutant. Proteins were quenched with TCA. All the mutants were incubated with SAM for three days before extensive dialysis. For Y76F and Y98A, SIC chromatography at m/z of 399.4 (SAM) and 385.4 (SAH) were shown.

a

b

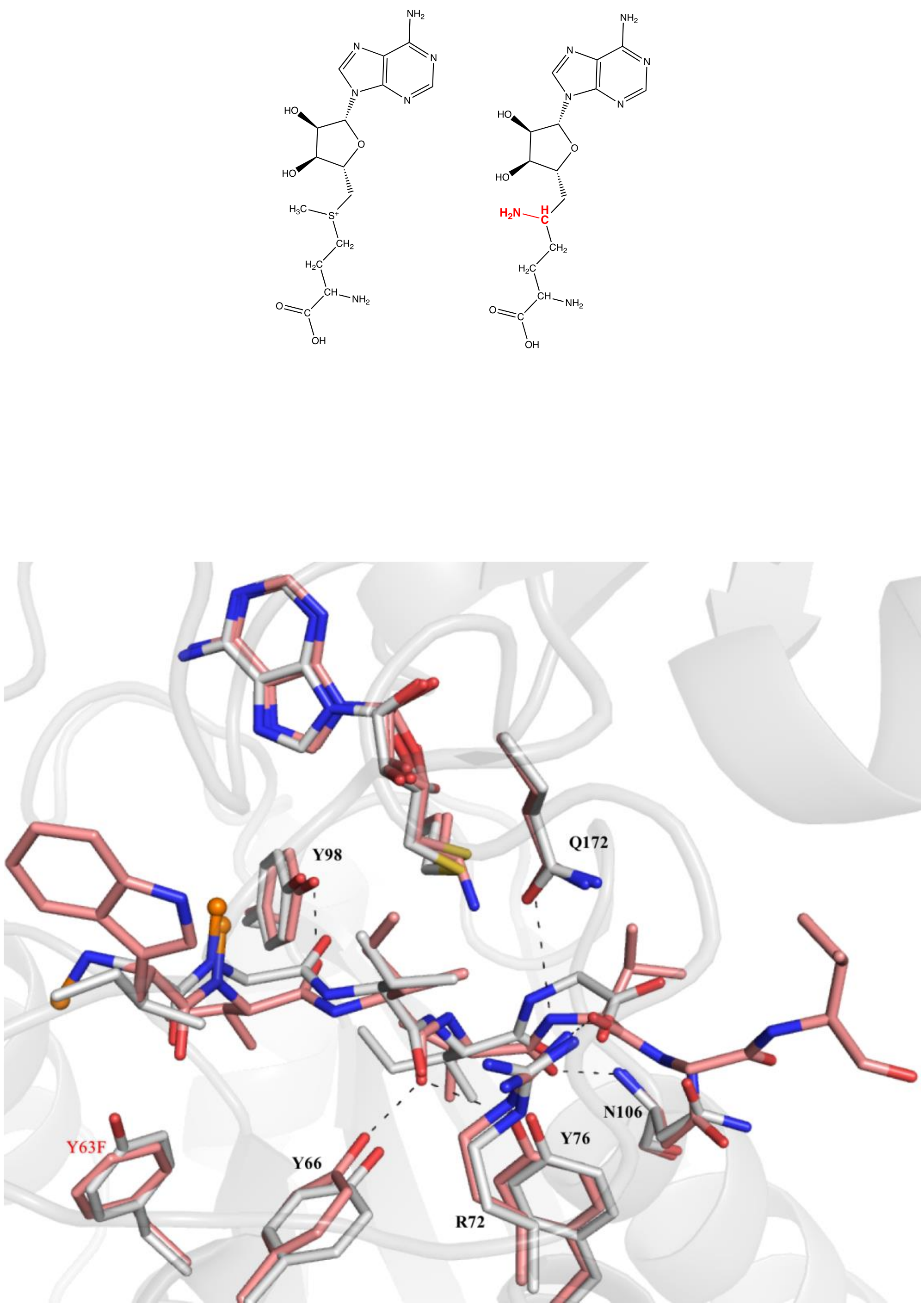

Fig. S4. Superimposition of OphAΔC6 structures. a Sinefungin on the right with red atoms is an inactive analogue of SAM shown on left. **b** OphAΔC6 Y63F-sinefungin superimposed with OphAΔC6-SAH, show essentially identical structures.

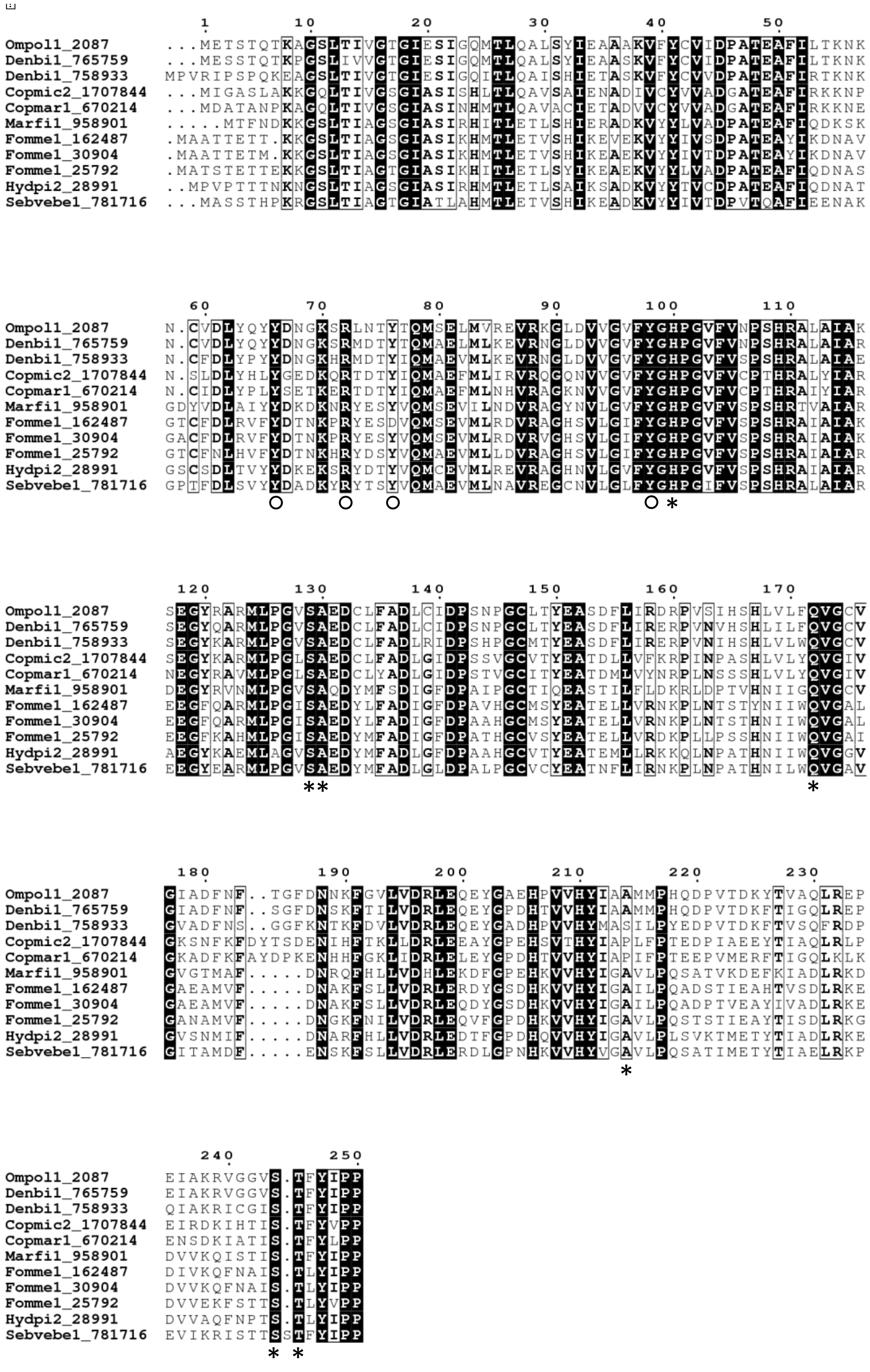

Fig. S5. Sequence alignment of the methyltransferase domain of OphA (Ompol1_2087). Shown are homologous putative methyltransferases found in basidiomycota using MultAlin (*72*) and ESPript 3.0 (*73*) Homologs were identified by BLASTP search of the JGI fungal genome database. Completely conserved residues are boxed in black and largely conserved residues (70 % +) are in open box. Asterisks and circles mark residues identified in this work as interacting with SAM and the substrate peptide, respectively. Full sequences and protein ID numbers are found on the JGI website (http://genome.jgi.doe.gov/programs/fungi/index.jsf).

a

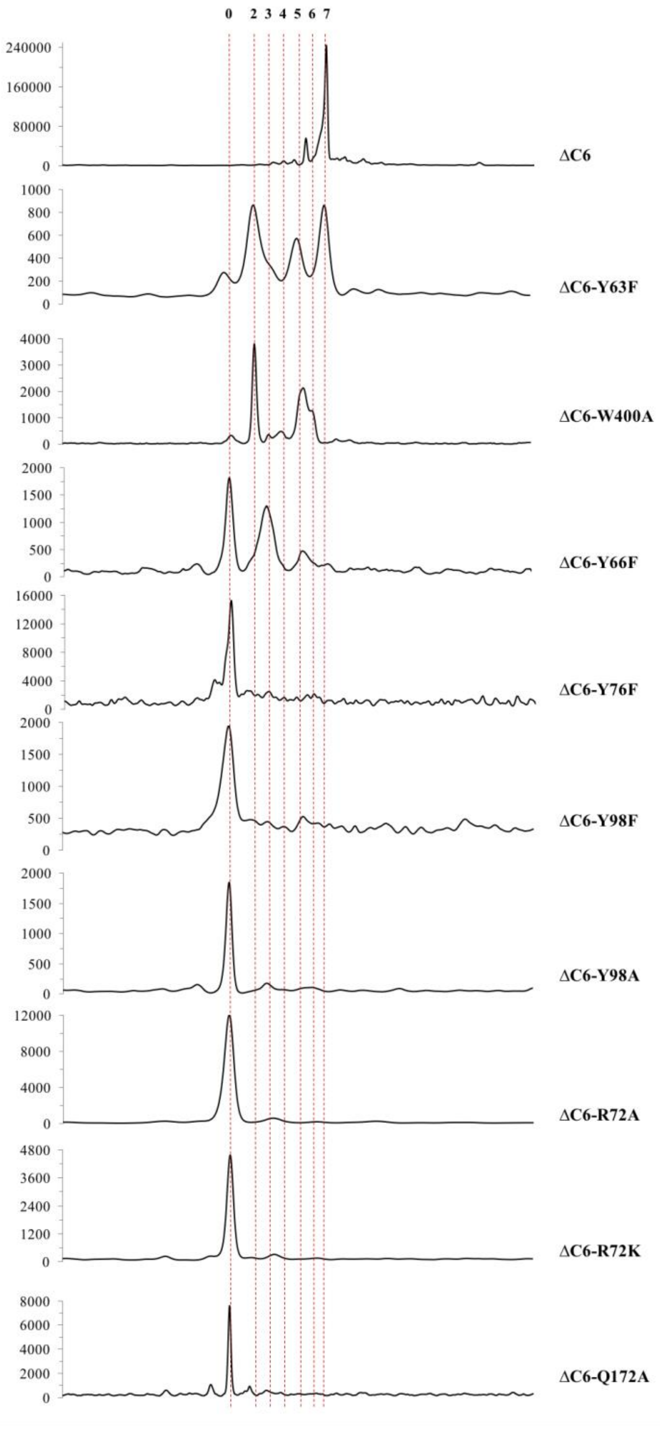

0 2 3 4 5 6 7
240000
160000
80000
0
ΔC6
1000
800
600
400
200
0
ΔC6-Y63F
4000
3000
2000
1000
0
ΔC6-W400A
2000
1500
1000
500
0
ΔC6-Y66F
16000
12000
8000
4000
0
ΔC6-Y76F
2000
1500
1000
500
0
ΔC6-Y98F
2000
1500
1000
500
0
ΔC6-Y98A
12000
8000
4000
0
ΔC6-R72A
4800
3600
2400
1200
0
ΔC6-R72K
8000
6000
4000
2000
0
ΔC6-Q172A

b

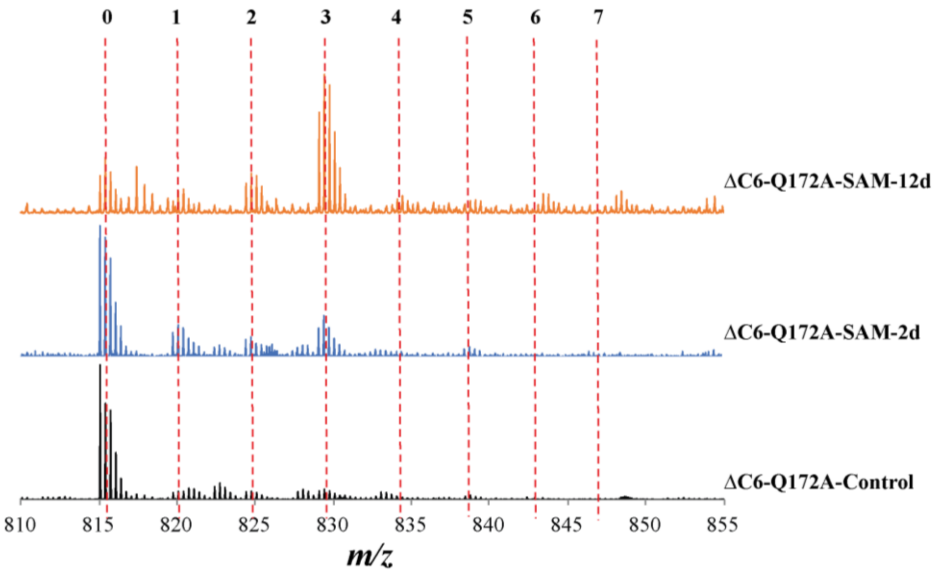

0
1
2
3
4
5
6
7
ΔC6-Q172A-SAM-12d
ΔC6-Q172A-SAM-2d
ΔC6-Q172A-Control
810
815
820
825
830
835
840
845
850
855
m/z

c

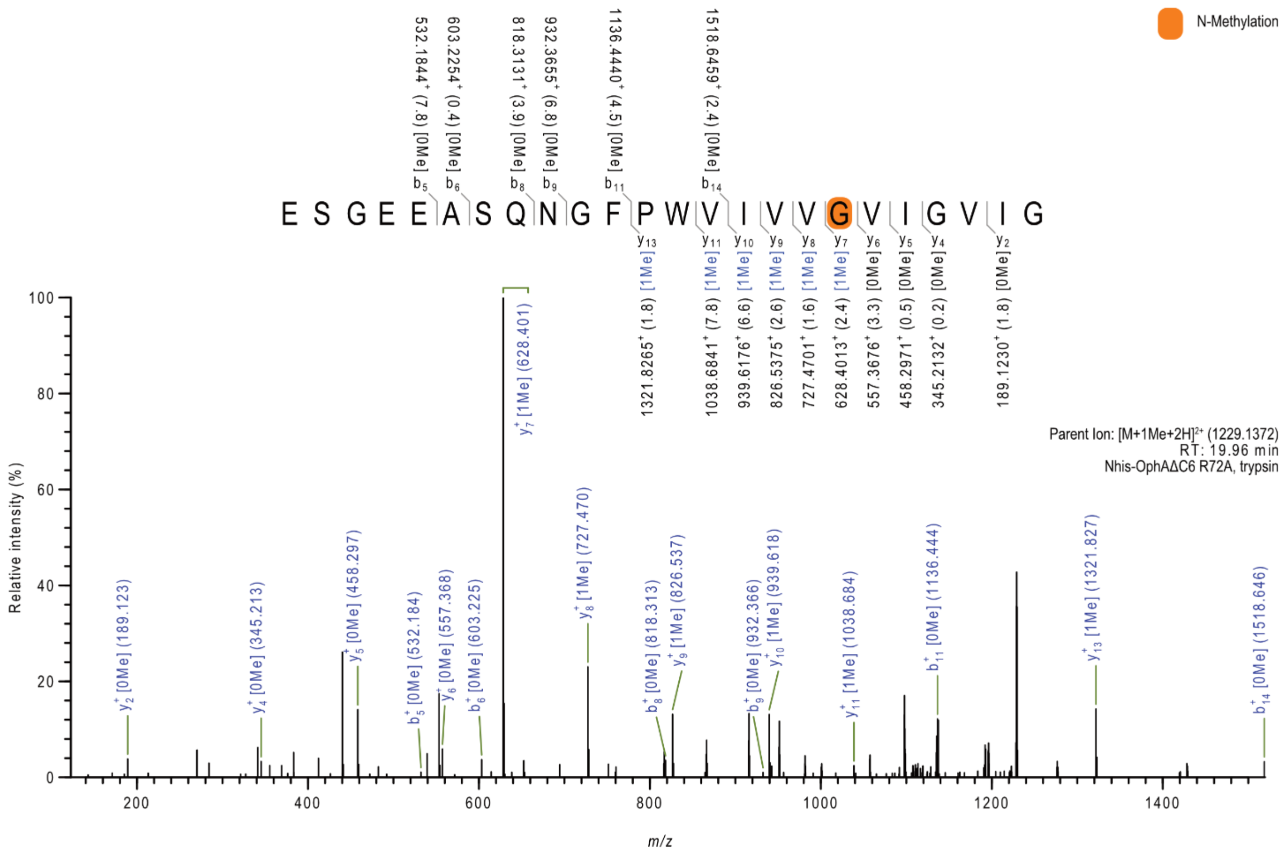

Fig. S6. Mass analysis of all mutants discussed in the manuscript. a The intact masses of the mutants designed to test the mechanism. The spectra were recorded for all protein after purification from a standard expression (16 °C for 20h with 0.2 mM IPTG except Y76F). Line for methylation are marked to aid interpretation; methylation was also confirmed by fragment analysis, ion counts are shown on y-axis, X-axis is molecular weight. b *In vitro* reaction of Q172A with SAM for 2d and 12d. Q172A shows much better activity than Y98A, Y76F, R72A and R72K. c. MS/MS fragmentation results for a singly-methylated OphA core only observed in trace amounts in an *in vitro* reaction of R72A run for 12d supplemented with 200 mM guanidine-HCl (method 2). Values in parentheses are errors in observed ions compared to theoretical masses, represented as ppm.

a

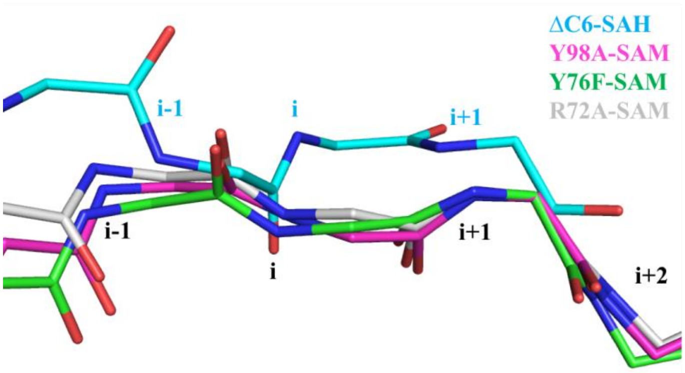

b

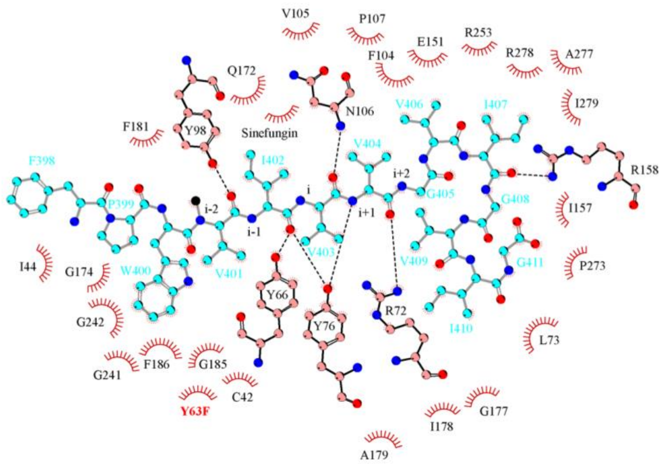

c

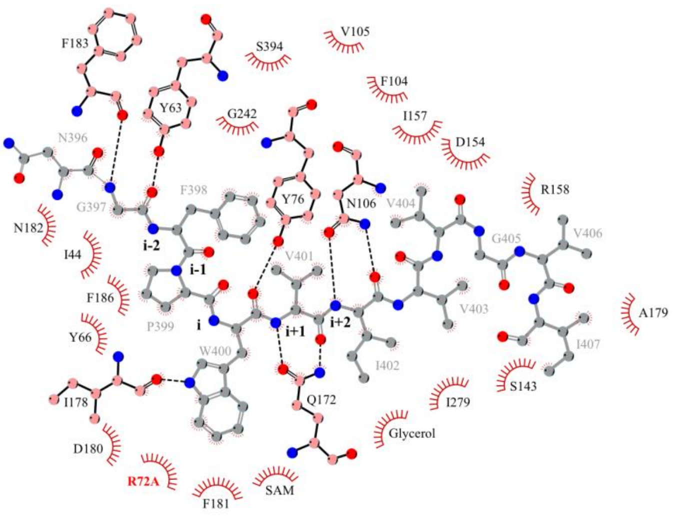

Fig. S7. All in vivo inactive mutants have similar inactive confirmations. a Superimposition of all three mutants structures (Y98A-SAM, Y76F-SAM and R72A-SAM) with wild type OphAΔC6-SAH. **b** LigPlot showing interactions of substrate peptide with protein and sinefungin in Y63F (active) structure. **c** LigPlot showing interactions of substrate peptide with protein and SAM in R72A-SAM (inactive) structure.

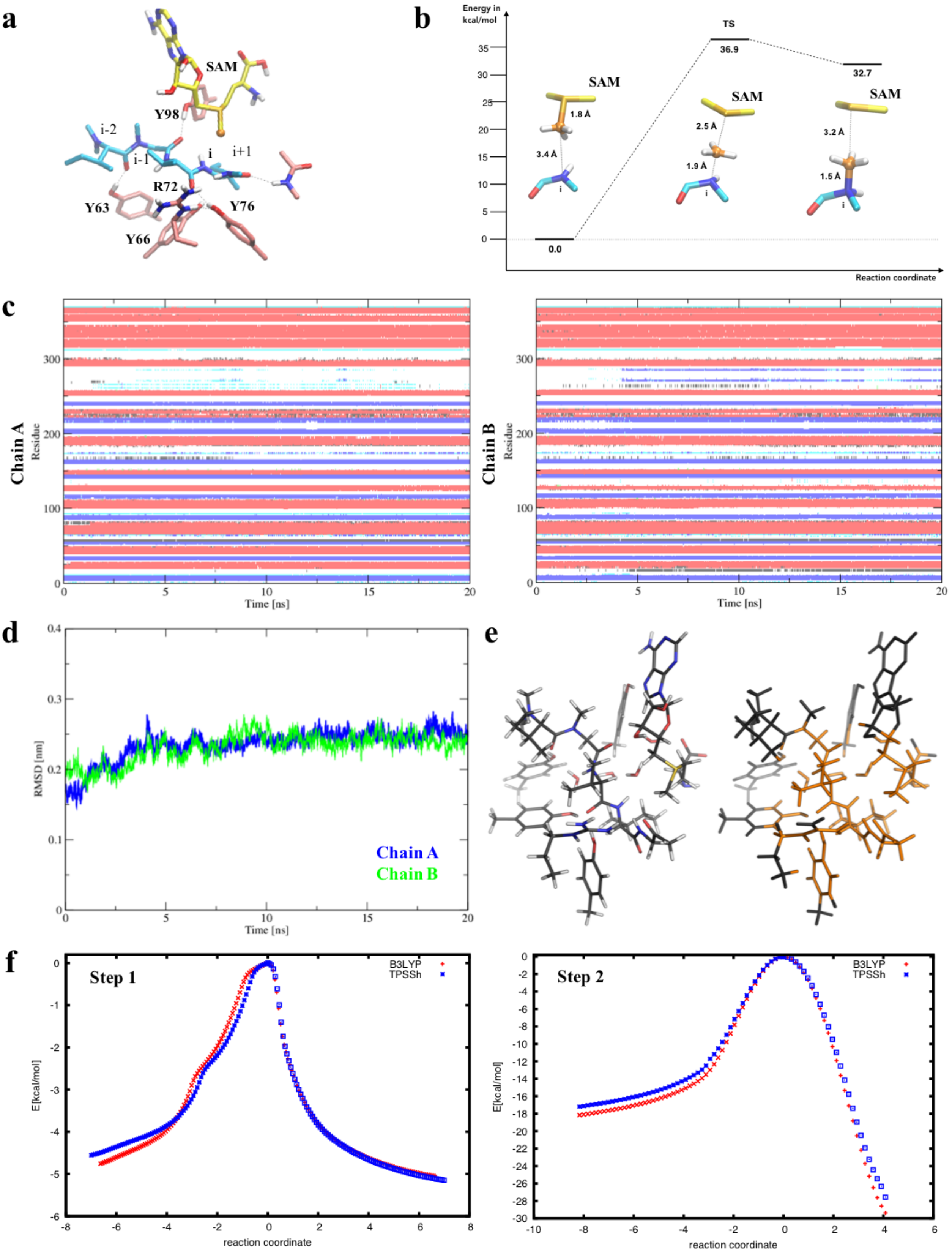

a
SAM
Y98
i-2
i-1
i
i+1
R72
Y63
Y76
Y66
b
Energy in kcal/mol
TS
36.9
32.7
0.0
SAM
1.8 Å
3.4 Å
2.5 Å
1.9 Å
3.2 Å
1.5 Å
Reaction coordinate
c
Chain A
Chain B
Residue
Time [ns]
d
RMSD [nm]
Time [ns]
Chain A
Chain B
e
f
Step 1
Step 2
B3LYP
TPSSh
E[kcal/mol]
reaction coordinate

Fig. S8. MD simulations and QM calculations. a Cutout of the crystal structure of OphAΔC6-SAH/M used as educt in the QM calculation of the direct S_N2 attack, optimized with the B3LYP functional. SAM is shown in yellow, the substrate in cyan and key residues in the binding pocket in pink. Apolar hydrogens are omitted for clarity. **b** Energy profile of the direct S_N2 attack obtained from QM calculations employing the B3LYP functional. A barrier of 36.9 kcal/mol was calculated. **c** Secondary structure elements of chain A (top) and chain B (bottom) in a 20-ns MD simulation of the dimer complex with SAM in explicit water. Due to the unresolved loop between the extreme C-terminus (substrate of the enzymatic reaction) and the rest of the protein, the extreme C-termini were treated as separate chains in the MD simulation. α-Helices are shown in red, 3_{10}-helices in black, π-helices in green, β-bridges in cyan, and β-strands in blue. **d** Atom-positional root-mean-square deviation (RMSD) of the backbone with respect to the minimized crystal structure for chain A (blue) and chain B (green) in a 20-ns MD simulation of the dimer complex with SAM in explicit water. Due to the unresolved loop between the extreme C-terminus (substrate of the enzymatic reaction) and the rest of the protein, the extreme C-termini were treated as separate chains in the MD simulation. **e** (Left): B3LYP-optimized structure of the educt used in the QM calculations. (Right): Same structure with atoms that were constrained during the geometry optimization and the location of the transition state shown in black. Atoms colored in orange are within 7 Å of the methyl group of SAM and retain all degrees of freedom during the QM optimization. **f** Intrinsic reaction coordinate (IRC) employing the B3LYP (blue) or TPSSh (red) functional for reaction step 1 (top) and step 2 (bottom).

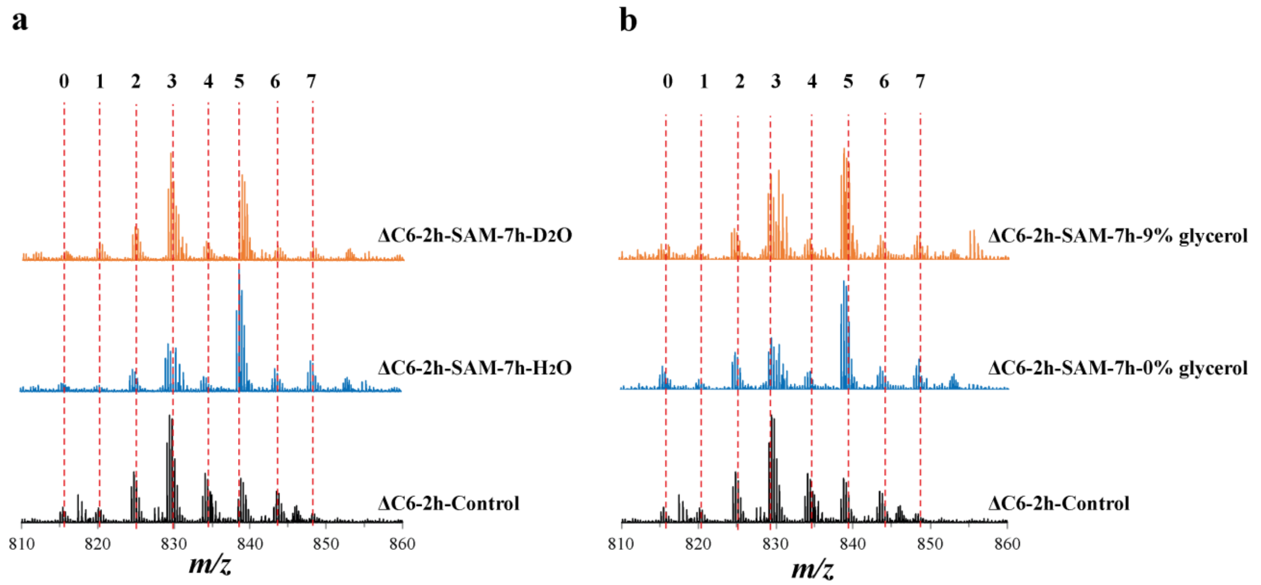

	H_2O	D_2O
k_{cat} (10^{-1}• h^{-1})	1.60 ± 0.03	0.52 ± 0.1
$k_{cat,H2O}/k_{cat,L2O}$	1.00 ± 0.02	3.1+0.5

	0% glycerol	9% glycerol
k_{cat} (10^{-1}• h^{-1})	1.11± 0.08	1.0± 0.1
$k_{cat,0\%Gly}/k_{cat,Gly}$	1.00 ± 0.07	1.1 ± 0.1

Fig. S9. Kinetic isotope effect and solvent viscosity effect studies of OphAΔC6-2h. a. Reactions using the same batch of protein were performed in 50 mM Tris buffer (pL 8.0). **b.** Solvent viscosity control experiments using another batch of proteins were performed in 50 mM Tris buffer pH 8.0, containing 0% glycerol and 9% glycerol as viscosigen (9% glycerol has same viscosity as D_2O). Solvent viscosity of D_2O has negligible effect on catalysis and primary solvent kinetic effect were observed, indicating proton transfer is important in catalysis. All the reactions were performed in replicates (n≥3, standard deviations were calculated) and one representative digestion spectrum was shown for ease. ΔC6-2h-control is protein incubated without SAM. *kcat* was calculated similarly to the pH-rate profile experiments.

Supporting Discussion

Computation

For the computation of reaction barriers, the energies of the educt, product and the transition state (TS) are required. Educt and product structures correspond to local minima on the potential energy hypersurface (PES), which can be located with geometry optimization algorithms. For this, a modification of the direct inversion of the iterative subspace (GDIIS) (*74*, *75*) method called GEDIIS (*76*) was used, which is the default algorithm in the Gaussian09 software package. In this method, the guess for the next optimization step is constructed by a linear combination of the previous steps. It is advantageous if the eigenvalues of the Hessian are small, i.e. the PES can be considered flat.

By contrast, a TS, which corresponds to a first-order saddle point on the PES, is characterized by having exactly one negative for its Hessian. This in turn corresponds to having exactly one imaginary frequency clearly defining one direction of downward curvature, which leads to the local minimum of the educt on one side of the TS and to the corresponding minimum of the product on the other side. Locating such a TS is a much more difficult task, since one cannot simply minimize the energy with respect to the nuclear coordinates as such a minimization would optimize a minimum on the PES with no negative eigenvalue by default. However, provided that the initial guess of the TS is already close to the actual TS, local methods like GDIIS can be applied.

Structural Biology

In order to test the assignment of the amino acid register, we re-refined the structure with -1 (Figure SD1a) and +1 (Figure SD1b) shifts register for the OphAΔC6-SAH/SAM complex. The register assigned in the manuscript fits the experimental density better than either alternative. Notably the main chain is well fitted in all three registers. This is the fundamental point, the substrate main chain is anchored by interactions, the side chains are not and we have been careful to focus on main chain interactions. This is not surprising as the protein processes multiple different side chains.

a

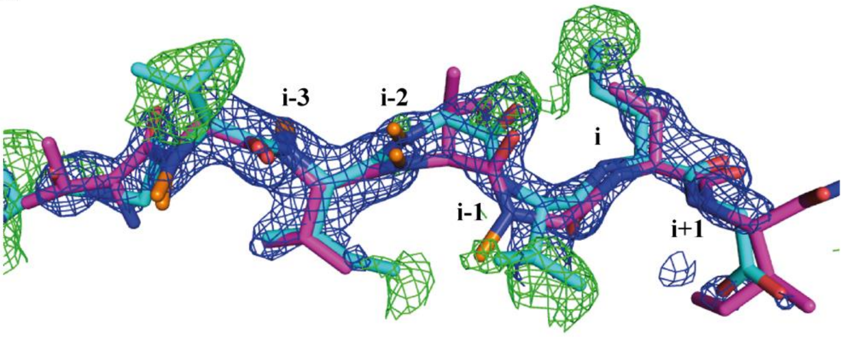

b

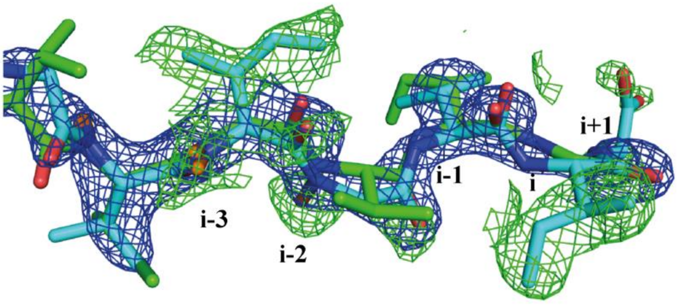

Fig. S10. Alternate register for substrate peptide. a Substrate peptide (C atom in purple) moves backwards by one residue (-1 position), i.e. Val 409 at position i. **b** Substrate peptide (C atom in green) moves forward by one residue (+1 position), i.e. Gly411 is at position i. In both figures the Fo-Fc difference map (2σ in green) and 2Fo-Fc map (1σ in blue) are shown for the alternative modelling of peptide for OphAΔC6-SAH/M. The final modelled substrate peptide (C atom in cyan) is shown for comparison.

Table S1. Crystallographic data.

	SeMet-OphAΔC18-SAM	OphAΔC18-SAM	OphAΔC18-SAH	OphAΔC6-SAM	OphAΔC6-SAH	OphAΔC6-Y63F-SAM	OphAΔC6-Y66F-SAM	OphAΔC6-R72A-SAM	OphAΔC6-Y76F-SAM	OphAΔC6-Y98A-SAM	OphAΔC6-R72A-SAH	OphAΔC6-Y76F-SAH	OphAΔC6-W400A-SAM	OphAΔC6-Y63F-Sinefungin	OphAΔC6-W400A-Sinefungin
**PDB ID**		**5N0O**	**5N0P**	**5N0X**	**5N0Q**	**5N0N**	**5N0R**	**5N0W**	**5N0T**	**5N0S**	**5N0U**	**5N0V**	**5N4I**	**6GEW**	**5OUF**
**Data collection**															
Space group	$P 2_1 2_1 2_1$	$P 2_1 2_1 2_1$	$P 2_1 2_1 2_1$	$P 2_1 2_1 2_1$	$P 2_1 2_1 2_1$	I 2 2 2	I 2 2 2	$P 2_1 2_1 2$	$P 2_1 2_1 2$	$P 2_1 2_1 2$	I 2 2 2	$P 2_1 2_1 2$	I 2 2 2	$P 3_2 2 1$	I 2 2 2
Cell dimensions															
a, b, c (Å)	74.6, 100.9, 123.3	74.6, 100.9, 123.3	74.8, 100.2, 124.5	72.7, 102.0, 121.3	72.4, 102.4, 115.3	87.3, 93.7, 165.3	86.7, 93.4, 165.4	163.1, 92.3, 85.7	166.1, 93.5, 86.6	163.8, 92.5, 85.3	86.1, 91.9, 164.4	163.7, 91.9, 85.6	85.9, 92.0, 162.2	89.8,89.8, 56.9	85.5. 91.9, 162.0
α, β, γ (°)	90.0, 90.0, 90.0	90.0, 90.0, 90.0	90.0, 90.0, 90.0	90.0, 90.0, 90.0	90.0, 90.0 90.0	90.0, 90.0, 90.0	90.0, 90.0, 90.0	90.0, 90.0, 90.0	90.0, 90.0, 90.0	90.0, 90.0, 90.0	90.0, 90.0, 90.0	90.0, 90.0, 90.0	90.0, 90.0, 90.0	90.0, 90.0, 120.0	90.0, 90.0, 90.0
Reflection (Unique)	1880030 (169405)	1880030 (169405)	280001 (51104)	452165 (104957)	148429 (33901)	371918 (67412)	464299 (84722)	426619 (96972)	935396 (129666)	553371 (93739)	673259 (74443)	730807 (100787)	621282 (85158)	467738 (43488)	527505 (70395)
Redundancy	11.1 (11.5)	11.1 (11.5)	5.5 (5.8)	4.3 (4.4)	4.4 (4.4)	5.5 (5.6)	5.5 (5.3)	4.4 (3.8)	7.2 (7.2)	5.9 (4.5)	9.0 (8.6)	7.3 (7.1)	7.3 (7.2)	10.8 (11.0)	7.5 (7.7)
Completeness (%)	100.0 (95.9)	100.0 (95.9)	99.9 (98.5)	99.8 (98.2)	99.2 (99.0)	99.5 (99.5)	98.0 (98.0)	98.7 (97.5)	100.0 (98.6)	98.4 (91.9)	99.9 (97.8)	99.9 (97.5)	98.6 (95.3)	100.0 (98.6)	100.0 (100.0)
I/σ(I)	18.6 (2.0)	18.6 (2.0)	20.9 (2.0)	12.3 (1.3)	11.9 (2.2)	24.0 (2.2)	27.6 (2.2)	17.4 (1.3)	14.1 (1.2)	13.0 (1.0)	10.8 (1.0)	12.6 (1.3)	14.9 (1.2)	12.3 (2.1)	14.1 (1.2)
R_{merge}[a]	0.070 (1.142)	0.070 (1.142)	0.035 (0.661)	0.045 (0.948)	0.074 (1.009)	0.032 (0.752)	0.025 (0.609)	0.071 (0.568)	0.054 (1.622)	0.098 (0.903)	0.083 (1.713)	0.065 (1.488)	0.045 (1.545)	0.077(1.017)	0.064 (1.730)
CC1/2 (%)		99.9 (75.1)	100.0 (79.0)	99.8 (63.6)	99.8 (86.1)	99.9 (79.1)	100.0 (84.2)	99.6 (76.8)	99.9 (56.5)	99.6 (65.7)	99.6 (51.5)	99.9 (57.8)	99.7 (60.5)	0.999 (0.556)	0.999 (0.470)
**Refinement**															
Resolution (Å)		61.64–1.44 (1.47–1.44)	78.07-2.16 (2.21-2.16)	78.04-1.67 (1.71-1.67)	61.34-2.40 (2.49-2.40)	50.54–1.76 (1.80-1.76)	81.29-1.61 (1.65-1.61)	49.8-1.93 (1.98-1.93)	83.0–1.78 (1.81–1.78)	85.3–1.95 (2.02–1.95)	49.9-1.68 (1.72-1.68)	85.6-1.91 (1.96-1.91)	75.9-1.59 (1.65-1.59)	77.8-2.10 (2.14-2.10)	49.55-1.70 (1.73-1.70)
R_{work} / R_{free}[b]		0.1714/0.1840	0.2001/0.2263	0.1959/0.2245	0.2248/0.2688	0.1881/0.2005	0.1777/0.1912	0.1937/0.2158	0.2118/0.2223	0.2053/0.2333	0.1972/0.2170	0.2183/0.2366	0.1822/0.2063	0.2348/0.2581	0.1651 /0.1915
No. atoms															
Overall		7100	5920	6472	5899	3462	3545	6711	6868	6895	3356	6602	3437	3001	3293
Protein		6197	5740	5969	5832	3155	3162	6117	6206	6303	3073	6115	3146	2948	3067
Ligands		94	60	60	52	48	49	60	54	68	30	52	56	53	43
Water		809	120	443	15	259	334	534	608	524	253	435	235	19	183
RMS (bonds) (Å)		0.010	0.010	0.010	0.007	0.009	0.009	0.008	0.010	0.009	0.007	0.007	0.007	0.008	0.008
RMS (angles) (°)		1.377	1.316	1.343	1.273	1.277	1.345	1.380	1.428	1.440	1.366	1.300	1.331	1.097	1.374
Ramachandran															
Favored (%)		96.86	96.91	96.73	6.59	95.32	95.29	95.62	94.95	95.88	96.12	96.34	95.06	96.73	95.54
Allowed (%)		2.86	2.54	2.99	3.14	4.16	3445	3.99	4.66	3.75	3.36	3.00	4.16	3.27	3.67
Outliers (%)		0.27	0.55	0.27	0.27	0.52	0.26	0.39	0.39	0.37	0.30	0.65	0.78	0	0.79
Average B-factor (Å²)															
Overall		27.0	73.7	40.2	92.9	45.0	40.0	46.2	38.4	45.8	43.8	41.9	42.8	90.3	35.8
Protein		25.7	74.2	40.2	93.3	44.1	39.3	46.0	37.9	45.7	43.5	41.9	42.4	91.0	35.3
Ligands		31.2	58.9	25.4	57.9	84.9	37.9	32.0	24.8	37.4	31.4	27.5	41.9	61.9	34.7
Solvent		36.5	55.3	43.1	58.3	48.7	47.2	49.8	44.6	49.0	48.2	43.7	47.8	70.1	44.7

Table S2. Occupancy of SAM/SFG and SAH in OphA variants. The first row shows the ratio of SAM/SFG to SAH estimated by HPLC in different variants after incubation with SAM or sinefungin and dialysis. Second row indicates the occupancy estimated by Phenix. The crystals were grown and handled in the presence of high concentration of SAM, sinefungin or SAH.

	**OphA-ΔC18**	**OphA-ΔC6-SAM**	**OphAΔC6-Y63F-50mM Sinefungin**	**OphAΔC6-Y63F-SAH**	**OphAΔC6-Y66F- SAM**	**OphAΔC6-W400A-sinefungin**	**OphAΔC6-R72A-SAM**	**Y76F-SAM**	**Y98A-SAM**
SAM(SFG):SAH<br><br>HPLC	1.0:0	0.1:0.9	0.6:0.4 (Solution)<br>0.7:0.3 (crystals)	0.1:0.9	0.1:0.9	0.7:0.3	0.8:0.2	No SAH detected	No SAH detected
SAM(SFG):SAH<br><br>Phenix	NA	0.3:0.7<br>(0.1:0.9)	0.8:0.2	NA (modeled SAH)	NA (Modeled SAH)	0.9:0.1	0.7:0.3<br>0.6:0.4	0.8:0.2<br>0.9:0.1	1.0:0<br>0.9:0.1
